# Prevention of Hypovolemic Circulatory Collapse by IL-6 Activated Stat3

**DOI:** 10.1371/journal.pone.0001605

**Published:** 2008-02-13

**Authors:** Jeffrey A. Alten, Ana Moran, Anna I. Tsimelzon, Mary-Ann A. Mastrangelo, Susan G. Hilsenbeck, Valeria Poli, David J. Tweardy

**Affiliations:** 1 Department of Pediatrics, Baylor College of Medicine, Houston, Texas, United States of America; 2 Breast Care Center and Department of Medicine, Baylor College of Medicine, Houston, Texas, United States of America; 3 Department of Medicine, Baylor College of Medicine, Houston, Texas, United States of America; 4 Department of Molecular and Cellular Biology, Baylor College of Medicine, Houston, Texas, United States of America; 5 Department of Genetics, Biology and Biochemistry, University of Turin, Turin, Italy; University of Arizona, United States of America

## Abstract

Half of trauma deaths are attributable to hypovolemic circulatory collapse (HCC). We established a model of HCC in rats involving minor trauma plus severe hemorrhagic shock (HS). HCC in this model was accompanied by a 50% reduction in peak acceleration of aortic blood flow and cardiomyocyte apoptosis. HCC and apoptosis increased with increasing duration of hypotension. Apoptosis required resuscitation, which provided an opportunity to intervene therapeutically. Administration of IL-6 completely reversed HCC, prevented cardiac dysfunction and cardiomyocyte apoptosis, reduced mortality 5-fold and activated intracardiac signal transducer and activator of transcription (STAT) 3. Pre-treatment of rats with a selective inhibitor of Stat3, T40214, reduced the IL-6-mediated increase in cardiac Stat3 activity, blocked successful resuscitation by IL-6 and reversed IL-6-mediated protection from cardiac apoptosis. The hearts of mice deficient in the naturally occurring dominant negative isoform of Stat3, Stat3β, were completely resistant to HS-induced apoptosis. Microarray analysis of hearts focusing on apoptosis related genes revealed that expression of 29% of apoptosis related genes was altered in HS vs. sham rats. IL-6 treatment normalized the expression of these genes, while T40214 pretreatment prevented IL-6-mediated normalization. Thus, cardiac dysfunction, cardiomyocyte apoptosis and induction of apoptosis pathway genes are important components of HCC; IL-6 administration prevented HCC by blocking cardiomyocyte apoptosis and induction of apoptosis pathway genes via Stat3 and warrants further study as a resuscitation adjuvant for prevention of HCC and death in trauma patients.

## Introduction

Traumatic injury is the leading cause of death for people in the first 4 decades of life in the United States [1] and other high-income countries [2] and the second leading cause of death after infectious diseases in low- and middle-income countries [2]. In those surviving the immediate lethal injury, the most important cause of death is hypovolemic circulatory collapse (HCC) [3]. The proposed mechanisms for development of HCC are cardiac dysfunction, abnormalities in vascular tone and capillary leak [4], [5]. Better delineation of the specific contributions of each of these abnormalities to HCC and an understanding of the molecular and cellular mechanisms underlying each abnormality has the potential to yield new strategies to prevent HCC.

To gain new mechanistic insights and to develop resuscitation strategies that prevent HCC, we developed a protocol in rats of minor trauma plus severe HS that reproducibly results in HCC. HCC in our model involves cardiac dysfunction secondary to cardiomyocyte apoptosis. IL-6 administration at the start of resuscitation prevents HCC, reverses cardiac dysfunction, improves mortality and eliminates cardiomyocyte apoptosis, at least in part, through activation of Stat3 which acts to normalize the shock-induced apoptosis pathway transcriptome. These findings support further investigation of IL-6, or other Stat3 activators, as an adjuvant for resuscitation of trauma patients with severe HS to prevent HCC.

## Methods

### Rat and mouse protocols for trauma plus hemorrhagic shock

These studies were approved by the Baylor College of Medicine Institutional Review Board for animal experimentation and conform to National Institutes of Health guidelines for the care and use of laboratory animals. Adult male Sprague-Dawley rats were obtained from Harlan (Indianapolis, IN). Stat3β homozygous-deficient (Stat3β^Δ/Δ^) mice were generated as described [6] and re-derived at Jackson labs. Pups from heterozygous matings were tailed and genotyped by PCR, as described, with minor modifications [6].

Rats (250–350 gm) were acclimatized and subjected to the sham or hemorrhagic shock (HS) protocols, as described [7], [8] with modifications. Blood (22.5 ml/kg body weight) was withdrawn into a heparinized syringe over 10 min then episodically thereafter to maintain the target MAP at 35 mmHg until blood pressure compensation failed. Blood was then returned as needed to maintain the target MAP. The amount of shed blood returned (SBR) defined 5 different levels of shock severity reflected in the duration of hypotension: 0% SBR (SBR0) represented the lowest level of shock severity (duration of hypotension, 78±2.5 minutes), 10% SBR (SBR10; duration of hypotension, 149±41.4 minutes), 20% SBR (SBR20; duration of hypotension, 165±32.7 minutes), 35% SBR (SBR35; duration of hypotension, 211±7.6 minutes), and 50% SBR (SBR50; duration of hypotension, 273±24.9 minutes). At the end of the hypotensive period, rats were fluid resuscitated over 30 min as described [7], [8]. Fluid resuscitation consisted of return of the remaining shed blood followed by a volume of Ringer's solution equal to 2 times the total shed blood volume. Ringer's solution contained a racemic mixture of L- and D-lactate. The MAP was observed at the end of resuscitation to assess for hypovolemic circulatory collapse (HCC) then the rats were humanely sacrificed 60 minutes after the start of resuscitation. HCC was defined as failing to achieve a MAP at the end of fluid resuscitation within the normal range of rats examined i.e. ≥72 mm Hg [the mean (94 mm Hg) minus two SD (11 mm Hg) of starting BP]. Where indicated, rats received 10 µg/kg of recombinant human IL-6 in 0.1 ml phosphate-buffered saline (PBS, pH 7.4) at the initiation of resuscitation or PBS alone. Sham rats were anesthetized and cannulated for 250 minutes but were not subjected to hemorrhage or resuscitation. One group of rats (UHS) was subjected to the most severe hemorrhagic shock protocol (50% SBR), but not resuscitated and kept at the target MAP (35 mmHg) for an additional 60 minutes (duration of hypotension = 336±10.3 minutes) before sacrifice.

Stat3β^Δ/Δ^ mice and wild-type littermate mice were subjected to a trauma/HS protocol [7], [8], which was similar to the rat protocol except that the target MAP in the mouse was 30 mm Hg and the duration of hypotension was 180 min in all mice. Sham mice were anesthetized and immobilized in a pair-wise fashion with HS mice and sacrificed at the same time as their HS companion.

Rat and mouse hearts were harvested immediately after sacrifice and snap frozen in liquid nitrogen.

### *In vivo* pharmacological inhibition of Stat3

To achieve pharmacological inhibition of Stat3 activity within the heart, rats were randomized to receive the G-quartet oligodeoxynucleotide (GQ-ODN) T40214 or non-specific (NS)-ODN (2.5 mg ODN/kg) complexed in polyethyleneimine by tail vein injection, as described [9], 24 hours prior to subjecting them to SBR50 protocol. Fluorescently labeled T40214 when delivered in this fashion was previously shown to accumulate within the cells of a variety of normal tissues including heart, liver and kidneys (Jing N, unpublished data 2003) as well as tumor tissue [9]; half-life of T40214 within tumors and surrounding normal tissue was ≥48 hr.

### Doppler ultrasound studies

A 20 MHz Doppler ultrasound probe custom-built was used for these studies. Rats were subjected to Doppler evaluation, 60 min after the start of resuscitation, as described [10].

### ELISA detection of cytoplasmic nucleosomes

Determination of cytoplasmic histone-associated DNA fragments (nucleosomes) was performed using the Cell Death Detection ELISA Plus Kit (Roche), following the instructions of the manufacturer and modified for detection of nucleosomes within tissues.

### TUNEL staining

TUNEL was performed using the ApopTag® Plus Peroxidase *In Situ* Apoptosis Detection Kit following the manufacturer's instruction.

### Electrophoretic mobility shift assay (EMSA)

EMSA was performed using heart tissue extracts from experimental groups as described [7]. The level of transcription factor activation was quantitated using PhosphorImager analysis combining the intensities of the Stat3 homodimer and Stat3/Stat1 heterodimer gel shift bands.

### Immunoblotting

Levels of Stat3 activation within the hearts of rats were assessed by immunoblotting using whole-tissue extracts of heart sections with mouse monoclonal antibody to Tyr705 phosphorylated (p)Stat3 (Cell Signaling Technology, Inc., Danvers, MA; 1:1000 dilution), as described [11]. The membrane was then stripped (using Restore™ Western Blot Stripping Buffer, PIERCE, Rockford, IL) and immunoblotting performed to detect total Stat3 protein in the whole tissue extracts of hearts using mouse IgG1 monoclonal antibody to Stat3 (BD Biosciences, Rockville, MD). Densitometry was performed using ImageQuant TL v2005 software (Amersham Biosciences, Buckinghamshire, England). Results are expressed as the ratio of pSTAT3 signal (after background signal subtraction) to total STAT3 signal (after background signal subtraction) for each sample.

### RNA isolation and oligonucleotide microarray hybridization

Total RNA was isolated from 4-5 micron cryotome slices of the frozen ventricular tissue using TRIzol® Reagent (Invitrogen, Carlsbad, California) single step RNA isolation protocol followed by purification with RNeasy® Mini Kit (QIAGEN, Hilden, Germany) as instructed by the manufacturer. Gene expression profiling was performed with the Affymetrix Rat Array RAE 230A following Affymetrix protocols used within the Baylor College of Medicine Microarray Core Facility.

### Microarray Analysis

We used Affymetrics GCOS (www.affymetrix.com), dChip [12] (www.dchip.org) and Array Analyzer (www.insightful.com) software packages for quality assessment and statistical analysis and annotation. Expression estimation and group comparisons were done with Array Analyzer.

Low-level analyses included background correction, quartile normalization and expression estimation using GCRMA [13]. One-way analysis of variance (ANOVA) with contrasts [14] was used for group comparisons on all genes and on the list of apoptosis pathway genes only. P values were adjusted for multiple comparisons using the BH (Benjamini-Hochberg) method [15], which provides a good balance between discovery of statistically significant differences and minimization of false-positive occurrences. The adjusted p-values are the false discovery rates (FDR), which represent the proportion of “significant” genes vs. those that are false or spurious “discoveries”. We used a FDR = 10% as cut-off, which is near the conservative end of the desirable range <50% [16].

### Quantitative reverse transcription-polymerase chain reaction (Q-RT-PCR)

To validate gene expression patterns of microarray hybridization, we performed Q-RT-PCR as described [8] for 4 genes (Mcl-1, Bcl-xL, Tob1 and Atf3).

### Statistical analysis

Statistical differences were analyzed using one-way ANOVA and the Student-Newman-Keuls test, Student's t test and Fischer's exact test where appropriate.

## Results

### HCC is accompanied by left ventricular contractile dysfunction and cardiomyocyte apoptosis

To gain insight into mechanisms of hypovolemic circulatory collapse (HCC) at the organ, cellular and molecular level, we established conditions for HCC in a rat model of trauma/hemorrhagic shock (HS) by increasing the duration of the hypotensive phase (Table 1). HCC was not observed with periods of hypotension up to 165 min (0 to 20% shed blood return; SBR0, SBR10 and SBR20, groups) but occurred in 20% of the SBR35 rats and 67% of SBR50 rats (hypotensive phase = 211±7.6 and 273±24.9 min, respectively).

**Table 1:** Incidence of HCC with increasing duration of the hypotensive phase.

Group (% SBR)^2^,^3^	Duration of hypotensive	% with HCC^1^
	Phase (min, mean±SD)	
0	78±2.5	0
10	149±41.4	0
20	165±32.7	0
35	211±7.6	20
50	273±24.9	67

1 Defined as failure to achieve a MAP at the end of resuscitation within the normal range i.e. ≥72 mm Hg [the mean (94 mm Hg) minus two SD (11 mm Hg) of starting BP].

2 SBR = shed blood return

3 n≥3

**Figure 1:**
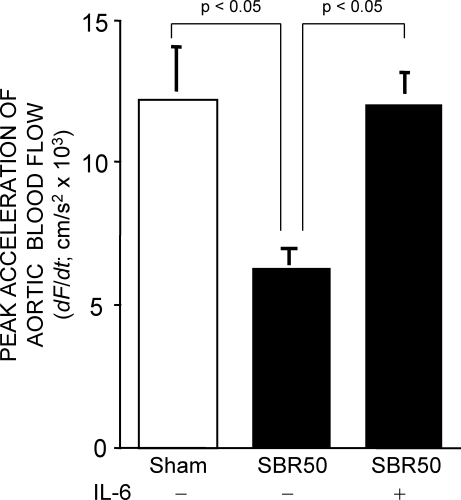
Effect of trauma/HS without or with IL-6 on peak acceleration of aortic blood flow. Rats were subjected to the sham or the SBR50 protocol; SBR50 rats were randomly assigned to receive either placebo or IL-6 at the start of resuscitation (n≥3 in each group). Sixty minutes after start of resuscitation or at the equivalent time point for shams, rats underwent Doppler studies of the ascending aorta. Data presented is the mean±SEM of peak acceleration of aortic blood flow; significant differences are indicated (one-way ANOVA followed by Student-Newman-Kuels test).

It has been demonstrated in non-rodent animal models that cardiac dysfunction contributes to HCC [4], [5]. To determine if this was the case in our rat model, we performed Doppler ultrasound studies of the ascending aorta of SBR50 rats (Figure 1); peak aortic acceleration has been demonstrated previously to be a good noninvasive index of cardiac contractile function in animals [10], [17]. Peak aortic acceleration in the SBR50 rats (6,974 cm/s^2^) was reduced by 50% compared to sham rats (14,061 cm/s^2^; p<0.05) indicating that HCC in rats was accompanied by left ventricular contractile dysfunction.

**Figure 2:**
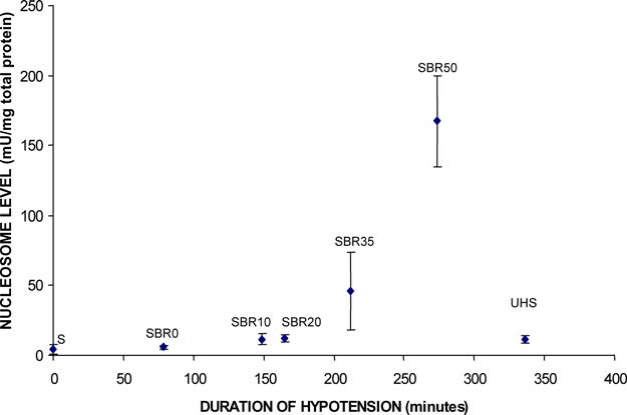
Effect of duration of hypotension and resuscitation on cardiac nucleosome levels. Rats (n≥3 in each group) were subjected to sham protocol (S) or the trauma/HS protocol with increasing severity of shock (SBR0, SBR10, SBR20, SBR35, and SBR50) as indicated followed by resuscitation. Hearts were harvested 60 minutes after the start of resuscitation. One group of rats (UHS) was subjected to the SBR50 protocol, but not resuscitated, rather, kept at the target MAP (35 mmHg) for an additional 60 minutes. Hearts were immediately harvested and snap frozen in liquid nitrogen. Nucleosome levels were measured in protein extracts of frozen sections of the heart and the results corrected for total protein and plotted as a function of the duration of the hypotensive period. Nucleosome levels increased exponentially with duration of hypotension (Pearson correlation coefficient = 0.764, p<0.0001, UHS group not included in the analysis).

**Figure 3:**
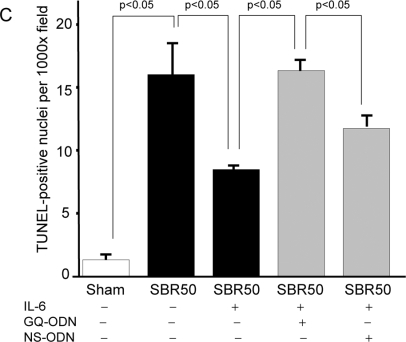
Effect of trauma/HS without or with IL-6 on cardiac apoptosis; impact of Stat3 inhibition on the IL-6 effect. Rats (n≥3 in each group) were randomly assigned to the sham or SBR50 protocol; SBR50 rats were randomized 24 hr prior to trauma/HS not to receive pretreatment or to receive pretreatment with GQ-ODN or non-specific (NS)-ODN by tail vein injection as indicated. At the start of resuscitation, rats in the first two SBR50 groups were randomized in a blinded fashion to receive either placebo or IL-6 at the start of resuscitation; each rat in the last two SBR50 groups received IL-6. In panel A, extracts of cryotome sections of flash-frozen ventricles obtained 60 minutes after the start of resuscitation were used to measure nucleosome levels by ELISA. Data presented are the mean±SEM of nucleosome levels (Units/mg protein); significant differences are indicated (one-way ANOVA followed by Student-Newman-Kuels test). In panel B, sections of fixed ventricles were stained using the TUNEL protocol; representative slides showing TUNEL-positive cells (arrows) are shown. In panel C, the number of TUNEL-positive nuclei was counted in twenty randomly chosen fields of each slide by an experienced microscopist blinded to the treatment the rats received. Data presented are mean number of TUNEL-positive nuclei per field±SEM for each group; significant differences are indicated (one-way ANOVA followed by Student-Newman-Kuels test).

Cardiomyocyte apoptosis has been observed in other settings of ventricular contractile dysfunction such as following myocardial infarction [18]. To assess if HCC is accompanied by cardiomyocyte apoptosis, we evaluated the hearts of rats for apoptosis using two assays—nucleosome ELISA and TUNEL staining. Nucleosomes levels (Figures 2 and 3A) were low in sham rat hearts (4.62±1.32 units/mg total protein) and did not increase in the hearts of SBR0, SBR10 or SBR20 rats. However, nucleosomes levels were increased nearly 10-fold over sham in SBR35 rats (45.64±27.8 units/mg total protein, p<0.01, ANOVA) and 36-fold over sham in SBR50 rats (167.3±24.9 units/mg total protein, p<0.01, ANOVA). The 3.7-fold increase in apoptosis in the SBR50 vs. SBR35 rats correlated well with the 3.3-fold increase in HCC in the SBR50 vs. SBR35 rats (Table 1). Results of TUNEL staining confirmed the nucleosome findings and indicated that cardiomyocyte nuclei were the major contributor to nucleosomes (Figure 3B and 3C).

To determine the contribution of resuscitation to cardiac apoptosis, we assessed nucleosome levels in the hearts of rats subjected to HS without resuscitation (UHS group, Figure 2; hypotensive phase = 336±10.3 min). The level of nucleosomes in the UHS group (8.1±2.3 units/mg total protein) was less than 18% of the value in the hearts of SBR35 rats (45.64±27.8 units/mg total protein, p<0.001, ANOVA) and less than 5% of the value in the hearts of the SBR50 group (167.3±24.9 units/mg total protein, p<0.001 ANOVA) indicating that resuscitation is required for virtually all HS-induced cardiac apoptosis. Results of TUNEL-staining confirmed the nucleosome findings; the number of TUNEL-positive nuclei/1,000X field in the hearts of UHS rats (2.2±0.5) was similar to sham rats (1.3±0.3; p>0.05). These results extend to the heart previous findings suggesting that resuscitation is needed for HS-induced liver, small intestine and lung apoptosis [19], [20] and suggest that interventions initiated at the start of resuscitation have the potential to prevent cardiomyocyte apoptosis along with HCC.

### IL-6 reverses HCC, ventricular contractile dysfunction and cardiomyocyte apoptosis

**Figure 4:**
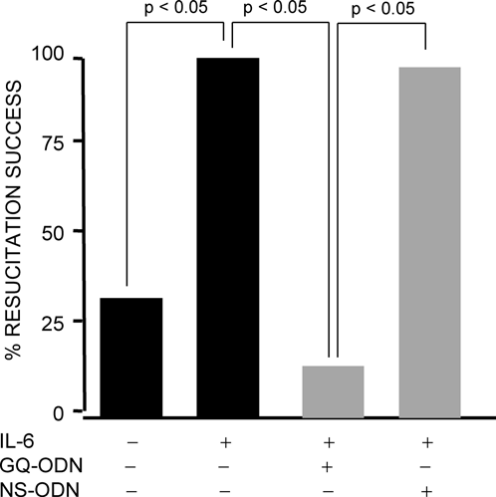
Resuscitation success following trauma/HS without or with IL-6 treatment; impact of Stat3 inhibition on the IL-6 effect. Rats were subjected to the 50% shed blood return protocol (SBR50) and randomized in a blinded fashion to receive either placebo (n = 6) or IL-6 (n = 7) at the start of resuscitation as indicated. Two additional groups of rats were randomly assigned 24 hr prior to SBR50 and IL-6 treatment to receive pretreatment with GQ-ODN or non-specific (NS)-ODN by tail vein injection as indicated (n≥7). Data presented is the percent resuscitation success for each group defined as achieving a MAP ≥mean MAP minus 2 SD of starting BP. Significant differences are indicated (Fisher's exact test).

We investigated whether administration of IL-6, a cytokine with anti-apoptotic properties, at the start of resuscitation to rats subjected to 50% SBR could prevent HCC (Figure 4). All SBR50 rats that received IL-6 (SBR50/IL-6 rats) were successfully resuscitated, compared to only 33% of placebo-treated SBR50 rats (p<0.05, Fisher's exact test). The post-resuscitation MAP for the placebo group decreased to 69±19 mm Hg from a baseline MAP of 86±5 mm Hg (p<0.05; Student's t-test). In contrast, the post-resuscitation MAP of the rats that received IL-6 (91±12 mm Hg) was essentially identical to their starting MAP (94±9 mm Hg).

To determine if prevention of HCC by IL-6 was a result of improved left ventricular contractile function, we determined peak aortic acceleration of SBR50/IL-6 rats and compared the results with placebo-treated SBR50 rats (Figure 1). Resuscitation with IL-6 completely reversed the trauma/HS-induced ventricular contractile dysfunction (p<0.05, one-way ANOVA with Student-Newman-Keuls test). To determine if cardiomyocyte apoptosis following trauma/HS is reversed by IL-6, we performed nucleosome ELISA assays (Figure 3A). Nucleosome levels in hearts from SBR50/IL-6 rats were reduced 94% compared to placebo-treated SBR50 rats (p<0.05, one-way ANOVA with Student-Newman-Keuls test). TUNEL assays of sections of rat hearts confirmed these findings (Figure 3B and 3C).

### IL-6 decreases mortality after trauma and HS

**Figure 5:**
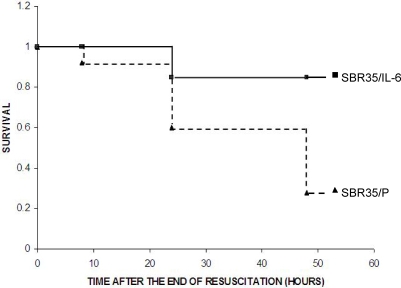
Effect of IL-6 on mortality following HS. Rats were subjected to the SBR35 protocol and randomized in a blinded fashion to receive either placebo (0.1 ml PBS; n = 25; SBR35/P; filled triangles) or IL-6 (10 µg/kg in 0.1 ml PBS; n = 26; SBR35/IL-6; filled squares) at the start of resuscitation. At the end of the resuscitation period, catheters were removed and anesthesia was reversed. Mortality was monitored and cumulative survival was plotted per Kaplan-Meier analysis (p<0.005).

Mortality following initially successful resuscitation from severe HS results from multi-organ dysfunction or failure [4], [5]. Following resuscitation, all SBR50 rats died regardless of whether or not they received IL-6 suggesting that the 50% SBR protocol caused failure of one or more vital organ(s) other than the heart that was not reversed by IL-6. To investigate the impact of IL-6 on mortality after trauma plus hemorrhage, SBR35 rats, which also experienced HCC and cardiomyocyte apoptosis (Table 1 and Figure 2), were randomized to receive either placebo or IL-6 (10 µg/kg) at the start of resuscitation (Figure 5). Similar to the effect of IL-6 treatment on HCC in the SBR50 group, all animals in the SBR35/IL-6 group were successfully resuscitated compared to 80% of rats in the placebo-treated SBR35 group (data not shown). Furthermore, mortality was reduced nearly 5 fold from 72% in placebo-treated SBR35 rats to 15.4% in SBR35/IL-6 group (p<0.005, Kaplan-Meier survival analysis).

### The ability of IL-6 to prevent HCC and cardiomyocyte apoptosis is Stat3 dependent

**Figure 6:**
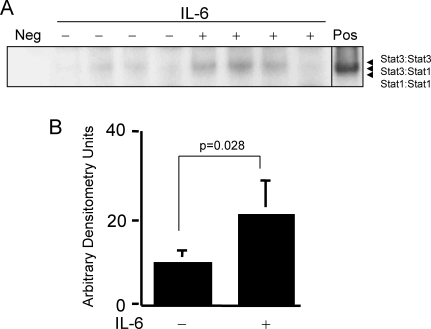
Effect of IL-6 on cardiac Stat3 activity. Rats were subjected to the SBR50 protocol and randomized in a blinded fashion to receive either placebo (0.1 ml PBS; −) or IL-6 (10 µg/kg in 0.1 ml PBS; +). Extracts (20 µg) of cryotome sections of flash-frozen ventricles were analyzed by EMSA; radiolabeled probe without cell extract (Neg) or an extract of cells previously shown to have Stat3 activity (Pos) were included as negative and positive controls, respectively. After separation, the gel was dried and autoradiographed (panel A) or subjected to phosphoimaging analysis (panel B). Data presented in panel B represents the mean±SD of the densitometry readings; significant differences are indicated (Student's t-test).

IL-6 activates Stat3, which has previously been demonstrated to activate the transcription of several anti-apoptotic genes and to contribute to apoptosis resistance in cancer cells (reviewed in [21]). To assess if the anti-apoptotic effect of IL-6 is mediated by Stat3 activation, we first determined if Stat3 is activated in the hearts of rats resuscitated with IL-6. Extracts of cryotome sections of the heart harvested 1 hour after IL-6 administration were examined by EMSA using radiolabeled duplex hSIE (Figure 6). Stat3-containing gel-shift bands, SIF-A and SIF-B (Stat3 homodimers and Stat3:Stat1 heterodimers, respectively) were more readily detected in heart extracts from SBR50/IL-6 rats than in heart extracts from placebo-treated SBR50 rats. Phosphoimaging analysis of Stat3-containing bands indicated that Stat3 DNA-binding activity is increased over two fold in the hearts of SBR50/IL-6 rats compared to placebo-treated SBR50 rats (p = 0.028, Student's t-test). Levels of phosphorylated Stat3 within hearts of SBR50/IL-6 rats also were increased, which confirmed the EMSA results (data not shown).

**Figure 7:**
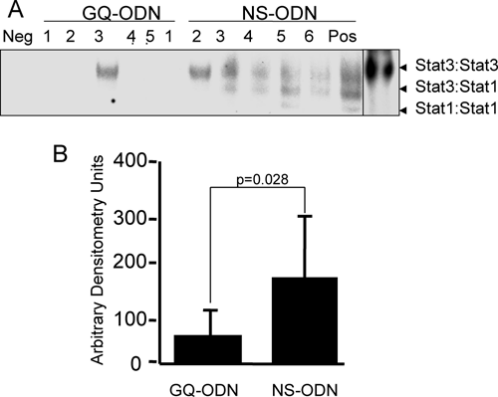
Effect of pretreatment with a Stat3 inhibitor on IL-6-induced Stat3 activity within the heart. Rats were pre-treated with GQ-ODN or NS-ODN and subjected to the SBR50 protocol and resuscitated with IL-6 (10 µg/kg in 0.1 ml PBS). Extracts of cryotome sections of flash-frozen ventricles were analyzed by EMSA; radiolabeled probe without cell extract (Neg) or an extract of cells previously shown to have Stat3 activity (Pos) were included as negative and positive controls, respectively. After separation, the gel was dried and autoradiographed (panel A) or subjected to phosphoimaging analysis (panel B). Data presented in panel B represents the mean±SD of the densitometry readings; significant differences are indicated (Student's t-test).

To determine if activation of Stat3 downstream of IL-6 was important for the improved resuscitation success observed in SBR50/IL-6 rats, we pre-treated rats 24 hr before SBR50 and IL-6 resuscitation with a G-rich, quartet forming oligodeoxynucleotide (GQ-ODN, T40214) that specifically binds to and inhibits the activity of Stat3 [9] or with a non-specific oligodeoxynucleotide (NS-ODN). Stat3 DNA-binding activity was reduced 3-fold within the hearts of SBR50/IL-6/GQ compared to SBR50/IL-6/NS rats (Figure 7). Levels of phosphorylated Stat3 within hearts of SBR50/IL-6/GQ rats also were decreased compared to SBR50/IL-6/NS rats, which confirmed the EMSA results (data not shown).

Resuscitation was successful in only 13% of SBR50/IL-6/GQ rats while resuscitation was successful in 94% of SBR50/IL-6/NS rats (p<0.05, Fisher's exact test; Figure 4). To determine if IL-6 activation of Stat3 was also important for the anti-apoptotic effects of IL-6 in SBR50, we examined the hearts of SBR50/IL-6 rats pre-treated with GQ-ODN vs. NS-ODN for nucleosome levels and TUNEL-positive nuclei. Nucleosome levels in the hearts of SBR50/IL-6/GQ rats (Figure 3A) were increased 10-fold compared to hearts from SBR50/IL-6 rats (p<0.05, one-way ANOVA with Student-Newman-Keuls test). Similarly, the number of TUNEL-positive nuclei in SBR50/IL-6/GQ rats was similar to that of placebo-treated SBR50 rats (Figure 3B and 3C). In contrast, nucleosome levels in the hearts of SBR50/IL-6/NS rats were virtually identical to the levels in the SBR50/IL-6 group (Figure 3A); the results of TUNEL staining in SBR50/IL-6/NS rats (Figure 3B and 3C) were consistent with the nucleosome results. Together these results indicated that Stat3 mediates a substantial portion of the effect of IL-6 in preventing cardiomyocyte apoptosis.

**Figure 8:**
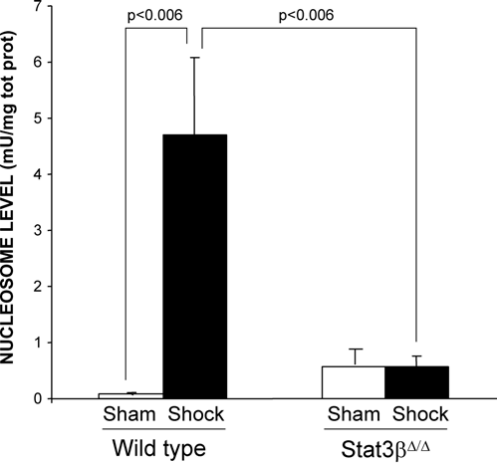
Effect of Stat3β ablation on trauma/HS-induced cardiac apoptosis. Stat3β homozygous-deficient (Stat3β^Δ/Δ^) mice and their littermate control wild type mice were subjected to the murine trauma/HS protocol or sham protocol and their hearts harvested 1 hr after the start of resuscitation. Nucleosome levels were measured in protein extracts of frozen sections of the heart and the results corrected for total protein. Data presented are the means±SEM of each group (n≥3). Significant differences are indicated (Student's t-test)

Two isoforms of Stat3 are expressed in all cells—α (p92) and β (p83)—both derived from a single gene by alternative mRNA splicing with Stat3α predominating. Stat3α functions as an oncogene [22] , in part, through inhibiting apoptosis, while Statβ antagonizes the oncogenic function of Stat3α [23]. While mice deficient in both isoforms of Stat3 are embryonic lethal at day 6.5 to 7 [24] and mice deficient in Stat3α die within 24 hr of birth, mice deficient in Stat3β have normal survival and fertility [6]. To further support the hypothesis that Stat3, in particular Stat3α, contributes to resistance to apoptosis within the heart in the setting of HS, we subjected Stat3β homozygous-deficient (Stat3β^Δ/Δ^) mice and their littermate control wild type mice to a severe HS protocol (target MAP 30 mm Hg for 5 hr) and examined their hearts for nucleosome levels 1 hr after the start of resuscitation (Figure 8). As expected, nucleosome levels in wild type HS mice were increased compared to wild type sham mice (p<0.006). In contrast, however, nucleosome levels in the hearts of Stat3β^Δ/Δ^ HS mice were reduced 88% compared to wild type HS mice and were similar to sham mice. These findings indicate that Stat3, in particular Stat3α, protects the heart from apoptosis in the setting of severe HS.

### Microarray analysis of the cardiac transcriptome focusing on differential expression of apoptosis pathway genes

The balance of pro- and anti-apoptotic proteins within the cell is regulated largely at the transcriptional level. Stat3 directly activates gene transcription and also interferes with the function of other transcription factors such as NF-κB [25], which previously has been shown to be activated following resuscitation from HS [7]. To identify apoptosis-related genes modulated within the hearts of animals suffering from HCC and to gain insight at the transcriptome level regarding how IL-6 protects against cardiomyocyte apoptosis, we performed Affymetrix oligonucleotide microarray analysis with RAE 230A chips. Initially, 12 chips were hybridized using mRNA isolated from 4 hearts each of sham, SBR50/P and SBR50/IL-6 rats. A separate experiment with 8 chips (4 in each group) was performed to compare SBR50/IL-6/GQ with SBR50/IL-6/NS groups. The 15,923 probesets on the RAE 230A chip represent 13,521 annotated genes or expressed sequence tags, including 859 apoptosis-related genes (Table S1, Supporting information files). The list of 859 apoptosis-related genes present on the REA 230A was created by combining gene lists obtained by querying annotation databases provided in Affymetrix, GeneSpring, and dChip derived from the Gene Ontology (GO) Consortium.

**Figure 9:**
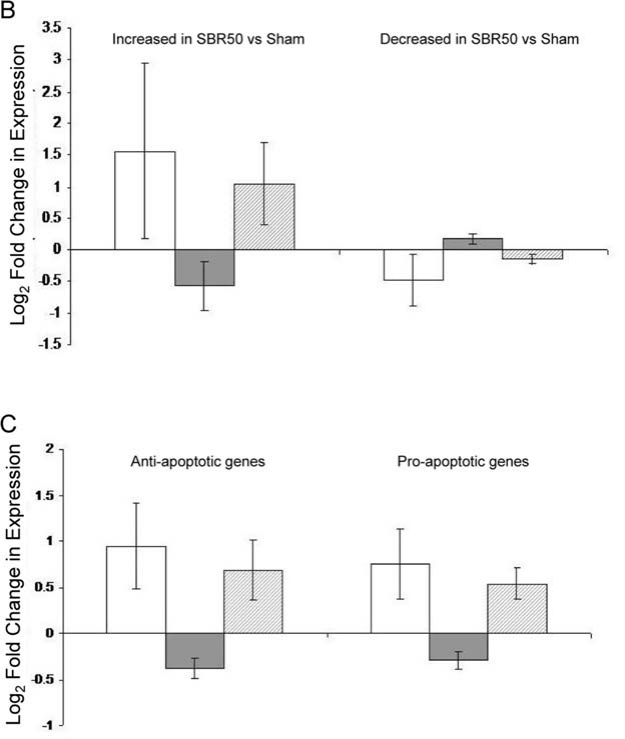
Effect of trauma/HS without or with IL-6 treatment on cardiac apoptosis-related gene expression; impact of Stat3 inhibition on the IL-6 effect. A. Heat map of apoptosis pathway genes whose expression is altered by trauma/HS. Rows in the heat map represent genes as listed in Table 1. Columns represent samples from the 3 groups examined in Experiment 1 (left panel) as indicated (S, Sham; P, placebo-treated SBR50; and I, SBR50/IL-6) and the 2 groups examined in Experiment 2 (right panel) as indicated (N, SBR50/IL-6/NS and G, SBR50/IL-6/GQ-ODN). Red indicates a level of expression above the mean expression of a gene within the experiment. White indicates a level of expression at the mean within the experiment while blue indicates a level of expression below the mean within the experiment. B and C. Changes in expression levels of apoptosis-related genes are shown comparing SBR50 vs. sham (open bars), SBR50/IL-6 vs. SBR50 (gray bars) and SBR50/IL-6/G vs. SBR50/IL-6/N (stippled bars). In panel B, the 196 apoptosis-related genes whose expression levels were changed in SBR50 vs. sham in the first microarray experiment were separated into those genes whose transcript levels were increased in SBR50 vs. sham (135 genes; left side of panel) and those whose transcript levels were decreased in SBR50 vs. sham (61 genes; right panel). In panel C, the 196 apoptosis-related genes whose expression levels were changed in SBR50 vs. sham in the first microarray experiment were separated into anti-apoptotic genes whose transcript levels were altered in SBR50 vs. sham (115 genes; left side of panel) and pro-apoptotic genes whose transcript levels were altered in SBR50 vs. sham (81 genes; right panel).

To identify genes differentially expressed among the three experimental groups (sham, SBR50, SBR50/IL-6), the data were filtered to remove genes with nearly uniformly low expression (absent on 80% of chips). Of the 859 apoptosis related genes present on the chips, 682 genes met the requirement of this filtering process and were included in the analysis (Table S1). One-way ANOVA (see Materials and Methods) was then performed which identified 201 apoptosis related genes with differential expression among the three groups at a False Discovery Rate (FDR) = 10%. Of the 201 apoptosis related genes whose expression was altered among the three groups, 196 were altered in the SBR50 vs. sham groups (Table S2 and Figure 9A). The majority (135 genes transcripts) were increased in SBR50 vs. sham by 1.1 to 75 fold while 61 gene transcripts were decreased in SBR50 vs. sham by 1.1 to 1.9 fold.

The overall effect of IL-6 administration on both sets of apoptosis-related gene transcripts was to “normalize” the alterations induced by trauma/HS (Figure 9B). Thirty-five (35) of the 135 gene transcripts that were increased in the SBR50 vs. sham groups were decreased significantly in the SBR50/IL-6 vs. SBR50 groups by 1.3 to 10.8 fold. Of the remaining 100 genes whose transcripts levels were increased, 84 were also decreased in the SBR50/IL-6 vs. SBR50 groups, although the decreases did not achieve statistical significance. Five (5) of the 61 gene transcripts that were decreased in the SBR50 vs. sham groups were increased significantly in the SBR50/IL-6 vs. SBR50 groups by 1.3 to 6.3 fold (Table S2). Of the remaining 56 genes whose transcript levels were decreased in the SBR50/IL-6 vs. SBR50 groups, 48 were increased although the increases did not achieve statistical significance. Thus, combining apoptosis-related gene transcripts that were both increased and decreased by trauma/HS (196 total), IL-6 treatment altered levels towards sham or normal in 172 (88%) of them.

Microarray analysis of hearts from SBR50/IL-6/GQ and SBR50/IL-6/NS rats identified 268 of the 682 apoptosis-related genes detected in the first microarray experiment whose expression was altered between the two groups (Table S2 and Figure 9A). One hundred and fourteen genes overlapped with the 201 genes identified within the first 3 groups. The overall effect of pharmacological inhibition of Stat3 in the apoptosis-related genes was to prevent the “normalizing” effect of IL-6 administration (Figure 9B). Specifically, of the 40 gene transcripts “normalized” statistically by IL-6 administration, 28 (70%) were significantly modulated within the SBR50/IL-6/NS rat hearts compared to the SBR50/IL-6/GQ rat hearts in the direction consistent with the hypothesis that IL-6 “normalized” SBR50/P-induced changes in gene transcript levels through activation of Stat3.

Q-RT-PCR was performed on a subset of the 201 genes whose expression was altered in the first microarray analysis and on Bcl-xL to establish the concordance rate of alteration in gene expression determined by microarrays with Q-RT-PCR, an alternative method for assessing levels of gene expression. The rate of agreement between microarrays and Q-RT-PCR in the direction of change in gene expression was 75%, which was similar to or better than reported by others [26].

## Discussion

We developed a hemorrhagic shock protocol in rats that models hypovolemic circulatory collapse (HCC). HCC in this model was accompanied by left ventricular contractile dysfunction secondary, at least in part, to cardiomyocyte apoptosis. HCC and cardiomyocyte apoptosis increased with increasing duration of hypotension. Apoptosis required resuscitation which provided an opportunity for therapeutic interventions. Administration of IL-6 at the start of resuscitation prevented HCC, improved left ventricular function, reversed cardiomyocyte apoptosis, reduced mortality and activated intracardiac Stat3. Pretreatment of rats with a specific GQ-ODN inhibitor of Stat3, T40214, blocked IL-6-mediated activation of intracardiac Stat3 and the ability of IL-6 to prevent HCC and cardiomyocyte apoptosis. The hearts of mice deficient in the naturally occurring dominant negative isoform of Stat3, Stat3β, were completely resistant to HS-induced apoptosis confirming a role for Stat3, particularly Stat3α, in this process. Microarray analysis of hearts focusing on apoptosis pathway genes revealed that expression of 29% of apoptosis-pathway genes was altered in SBR50 vs. sham rats. The overall effect of IL-6 treatment was to normalize the expression of these genes, while T40214 pretreatment prevented their normalization by IL-6. Thus, cardiomyocyte apoptosis occurs in our model of severe HS and contributes to left ventricular dysfunction and HCC; the cardioprotective effects of IL-6 in HCC are mediated in part by Stat3 through its ability to prevent cardiomyocyte apoptosis and “normalize” the shock-induced, apoptosis pathway transcriptome.

While left ventricular dysfunction in severe HS and its physiological details were reported over half a century ago [4], its cellular and molecular basis has remained incompletely defined. Cardiomyocyte apoptosis has been well described in other cardiac insults including rodent coronary artery occlusion models [18]. Furthermore, transgenic over-expression of the anti-apoptotic protein Bcl-2 within cardiomyocytes [18] resulted in decreased cardiac apoptosis and improvement in ventricular function. While apoptosis has been demonstrated in multiple organs after HS, it has not been previously demonstrated to occur within the heart following this insult. We observed a striking correlation between cardiomyocyte apoptosis and development of HCC in our rat model (Table 1 and Figure 3). Apoptosis was not detected within the hearts of SBR0, SBR10 and SBR20 rats in which HCC did not occur; rather, it was detected only in SBR35 and SBR50 rats that experienced HCC. In addition, the 3.7-fold increase in cardiac apoptosis in SBR50 vs. SBR35 rats was matched by a 3.4-fold increase in the incidence of HCC in SBR50 vs. SBR35 rats. In addition, IL-6 administration, which prevented HCC, also prevented cardiomyocyte apoptosis along with left ventricular dysfunction. Together, these findings strongly support the hypothesis that cardiomyocyte apoptosis contributes to HCC and left ventricular dysfunction following trauma/HS.

Binding of IL-6 to its receptor activates Stat3 (reviewed in [27]). *In vitro* and *in vivo* findings of others have provided evidence that Stat3 protects against cardiomyocyte apoptosis in some cardiac insults including ischemia-reperfusion injuries [28], [29]. However, the role of cardiac Stat3 in HS has not been investigated. We observed an increase in Stat3 activity within the hearts of rats that received IL-6 compared to placebo-treated rats. Furthermore, we determined that pre-treatment of rats with a specific GQ-ODN inhibitor of Stat3 blocked the IL-6-mediated increase in intra-cardiac Stat3 activity along with the ability of IL-6 to prevent HCC and to inhibit cardiomyocyte apoptosis. These findings indicate that Stat3 mediates the cardioprotective effects of IL-6, in large measure. The finding that the hearts of mice deficient in the naturally occurring dominant-negative isoform of Stat3, Stat3β, were completely resistant to HS-induced apoptosis lends strong support to this conclusion.

The functions of Stat3 identified to date involve regulation of gene transcription. The best-characterized transcriptional function of Stat3 is activation of gene transcription through binding to acute phase response elements (APRE) within gene promoters (reviewed in [21]). However, more recently, Stat3 also has been demonstrated to selectively inhibit transcription of specific genes such as Fas and the endothelial isoform of nitric oxide synthase (eNOS) [30], [31]; inhibition, in some cases, is mediated by cooperation with or interference of other transcription factors, notably c-Jun and NF-κB, respectively [25], [30].

Whether a cell survives or undergoes apoptosis in response to injury or stress is determined by the balance of anti- and pro-apoptotic proteins within the cell. The level of anti- and pro-apoptotic proteins is determined largely at the transcriptional level. To assess which apoptosis-related gene transcripts are altered within the hearts of rats by trauma/HS that lead to apoptosis and whether or not IL-6 alters this pattern of expression thereby tipping the balance away from apoptosis, we performed a global assessment of apoptosis-related gene transcript levels using microarray analysis. We identified 859 apoptosis-related genes contained within the RAE 230A GeneChip (Table S1) of which 682 were detectably expressed on 20% or more of the chips hybridized with heart mRNA. One hundred and ninety-six of these 682 genes (29%) were altered in the hearts of SBR50 vs. sham rats; this percentage of apoptosis genes altered was 2.1 fold greater than all the genes altered between SBR50 and sham hearts underscores the importance of the apoptosis gene pathway in our trauma/HS model at the transcriptome level (probability of equality of these proportions is less than 0.001).

Remarkably, the total number of genes differentially expressed in the SBR50/IL-6 vs. SBR50 group was 47 of which 44 (95%) belong to the apoptosis-related gene set (Table S1). Thus, the striking effect of IL-6 on cardiomyocyte apoptosis is reflected in its effect on the transcriptome being predominantly within the apoptosis-related gene set. Importantly, the predominant alteration found within 40 of these 44 genes altered in SBR50/IL-6 vs. SBR50 was “normalization” i.e. a return of their mRNA levels to sham levels. These findings strongly support the hypothesis that trauma/HS leads to an alteration in the balance of anti- and pro-apoptotic proteins that favors apoptosis and that IL-6 moves the balance away from apoptosis and back to “normal” or sham.

The findings from the second microarray analysis of hearts from SBR50/IL-6/NS and SBR50/IL-6/GQ rats indicates that Stat3 activation downstream of IL-6 is responsible, in large measure, for this normalizing effect of IL-6 upon the apoptosis transcriptome. Of the 40 genes that were normalized by IL-6 administration, 28 (70%) were modulated within the NS-ODN vs. GQ-ODN groups in the direction consistent with the hypothesis that Stat3 mediated the normalizing effect. Of these 28 genes, 27 genes were normalized by virtue of being decreased in the NS-ODN vs. the GQ-ODN group. Thus, the major effect of IL-6-activated Stat3 appears to be suppression of apoptosis related gene induction. The ability of Stat3 to inhibit gene transcription previously has been proposed to explain the anti-inflammatory effects of Stat3 activated downstream of IL-10 [32]. Our findings indicate that this is also the major way that IL-6-activated Stat3 modulates apoptosis following trauma and severe HS.

To determine which subset of apoptosis-related gene transcripts—anti-apoptotic, pro-apoptotic or both—were altered by trauma/HS and subsequently normalized by IL-6-activated Stat3, we analyzed each subset of transcripts separately (Figure 9C). Interestingly, gene transcript levels within both anti- and pro-apoptotic subsets were increased by trauma/HS; furthermore, transcripts within both subsets were normalized by IL-6, an effect that was reversed by pre-treatment with the Stat3 inhibitor, T0214. Since normalization by reduction of anti-apoptotic gene transcripts is very unlikely to protect from apoptosis, this analysis strongly suggests that trauma/HS results in cardiomyocyte apoptosis through upregulation of pro-apoptotic genes and that IL-6-activated Stat3 protects cardiomyocytes from apoptosis by inhibiting their upregulation,.

Two pro-apoptotic genes whose expression is consistent with this hypothesis are *Nr4a3* and *Nr4a1* (top two pro-apoptotic genes in Group 1A, Figure 7 and Table S2), also known as *Nor1* and *Nur77*, respectively. *Nr4a3/Nor1* is thought to be an important regulator of cellular mechanisms including apoptosis. It was originally identified from primary culture rat forebrain cells undergoing apoptosis [33]. Overexpression of *Nr4a3/Nor1* in thymocytes results in their apoptosis [34]. *Nr4a1/Nur77* is an immediate early response gene expressed in a wide variety of metabolically active tissues, including the heart; *Nr4a1/Nur77* encodes an orphan nuclear receptor with pleiotropic physiological roles including apoptosis induction (reviewed in [35]). Studies examining the effects of trauma/HS in *Nr4a3/Nor1*- and *Nr4a1/Nur77*-deficient mice are underway to determine if they are protected from HS-mediated cardiac apoptosis.

Our findings raise the possibility that IL-6 administration may be a useful adjuvant for resuscitation of trauma patients suffering from severe HS to prevent development of HCC. Recombinant human IL-6 has been given by subcutaneous and intravenous injection to human subjects as part of Phase I/II clinical trials performed to examine its potential thrombopoietic effects following cancer chemotherapy. Our findings coupled with previous reports that IL-6 was well tolerated in volunteers in doses up to 30 µg/kg/day for 7 days [36] suggest that a single intravenous bolus of IL-6 administered at the start of resuscitation of hypotensive trauma patients deserves further study to assess whether or not it prevents HCC and reduces mortality.

## Supporting Information

Table S1 Supplemental Table 1
(1.29 MB DOC)

Table S2 Supplemental Table 2
(0.38 MB DOC)
