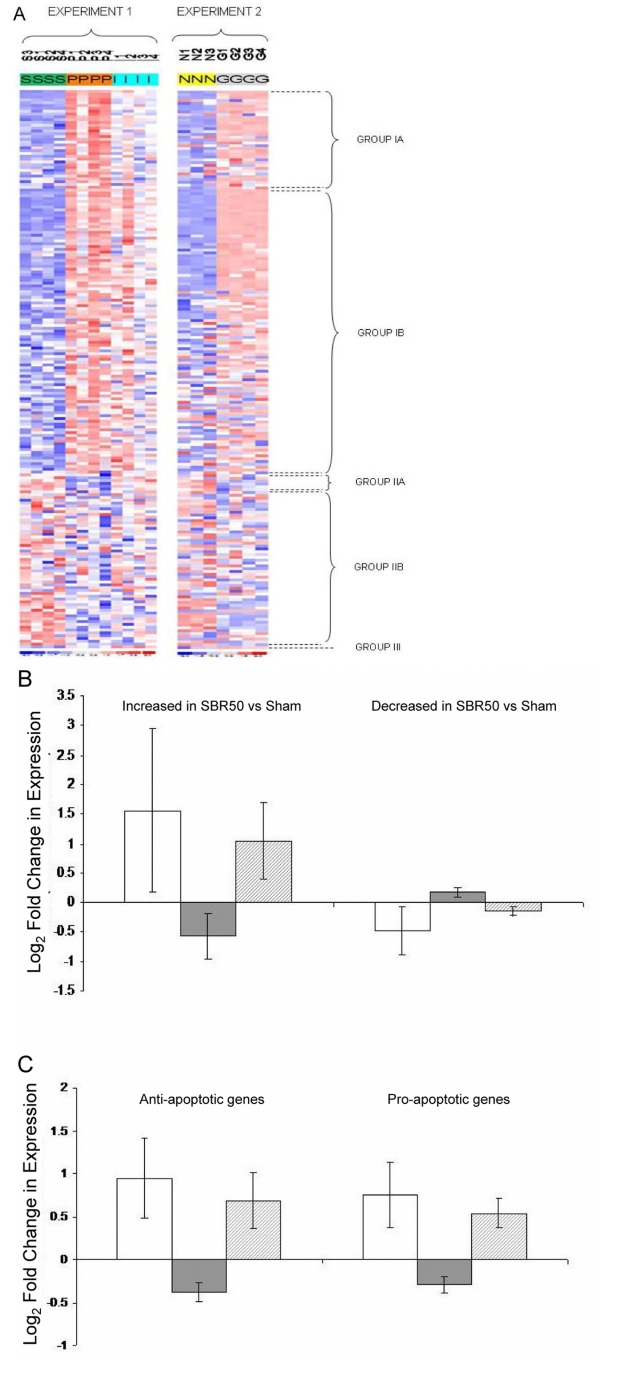# Correction: Prevention of Hypovolemic Circulatory Collapse by IL-6 Activated Stat3

**DOI:** 10.1371/annotation/c13f83c0-dc7e-4e5f-9b85-2bd041d3a6d8

**Published:** 2008-06-02

**Authors:** Jeffrey A. Alten, Ana Moran, Anna I. Tsimelzon, Mary-Ann A. Mastrangelo, Susan G. Hilsenbeck, Valeria Poli, David J. Tweardy

Figure 9A does not appear. Please view the entire Figure 9 here: 

**Figure:**