# Supplementary material for: Prevention of Hypovolemic Circulatory Collapse by IL-6 Activated Stat3
Source: PLoS One. 2008 Feb 13;3(2):e1605. doi: 10.1371/journal.pone.0001605 (PMC2225503; doi:10.1371/journal.pone.0001605)
Supplement: Table S1 — Supplemental Table 1 (1.29 MB DOC) [file pone.0001605.s001.doc]

**Table S1**. Apoptosis-related genes examined in the microarray experiments.

| **#** | **Accession #** | **NAME** | **Symbol** | **Present** | **Exp. 1** | **Exp. 2** |
| --- | --- | --- | --- | --- | --- | --- |
| 1 | BF417479 | 24-dehydrocholesterol reductase | Dhcr24 | Yes | Yes | No |
| 2 | NM_022225 | 5-hydroxytryptamine (serotonin) receptor 1B | Ets1 | Yes | Yes | No |
| 3 | NM_030870 | 8-oxoguanine DNA-glycosylase 1 | Ogg1 | Yes | No | No |
| 4 | NM_020306 | a disintegrin and metalloproteinase domain 17 (TNFA converting enzyme) | Adam17 | Yes | No | Yes |
| 5 | NM_131911 | acidic nuclear phosphoprotein 32 family, member B | Anp32b | Yes | No | No |
| 6 | NM_012912 | activating transcription factor 3 | Atf3 | Yes | Yes | Yes |
| 7 | BM391471 | activating transcription factor 5 | Atf5 | Yes | No | Yes |
| 8 | NM_019361 | activity regulated cytoskeletal-associated protein | Arc | Yes | No | Yes |
| 9 | AI600029 | activity-dependent neuroprotective protein | Adnp | Yes | Yes | Yes |
| 10 | NM_017155 | adenosine A1 receptor | Adora1 | Yes | No | Yes |
| 11 | AF228684 | adenosine A2a receptor | Adora2a | Yes | Yes | No |
| 12 | NM_012896 | adenosine A3 receptor | Adora3 | Yes | Yes | Yes |
| 13 | NM_031006 | adenosine deaminase, RNA-specific | Adar | Yes | No | Yes |
| 14 | AW523747 | adhesion molecule with Ig like domain 2 | Amigo2 | Yes | No | No |
| 15 | U07126 | adrenergic receptor, alpha 1a | Adra1c | No | N/A | N/A |
| 16 | AY057895 | adrenergic receptor, beta 2 | Adrb2 | Yes | No | No |
| 17 | NM_012715 | adrenomedullin | Adm | Yes | Yes | Yes |
| 18 | NM_134326 | albumin | Alb | Yes | No | No |
| 19 | NM_022407 | aldehyde dehydrogenase family 1, member A1 | Aldh1a1 | Yes | Yes | No |
| 20 | NM_012498 | aldo-keto reductase family 1, member B4 (aldose reductase) | Akr1b4 | Yes | No | No |
| 21 | NM_017196 | allograft inflammatory factor 1 | Aif1 | Yes | No | Yes |
| 22 | NM_012493 | alpha-fetoprotein | Afp | No | N/A | N/A |
| 23 | NM_012892 | amiloride-sensitive cation channel 1, neuronal (degenerin) | Accn1 | No | N/A | N/A |
| 24 | BM986220 | amyloid beta (A4) precursor protein | App | Yes | No | Yes |
| 25 | NM_053957 | amyloid beta (A4) precursor protein-binding, family B, member 3 | Apbb3 | Yes | No | No |
| 26 | NM_053957 | amyloid beta (A4) precursor protein-binding, family B, member 3 | Appb3 | No | N/A | N/A |
| 27 | U90829 | amyloid beta precursor protein binding protein 1 | Appbp1 | Yes | No | No |
| 28 | NM_012502 | androgen receptor | Ar | Yes | No | No |
| 29 | AF275151 | androgen receptor-related apoptosis-associated protein CBL27 | Cbl27 | Yes | No | No |
| 30 | BI275292 | angiopoietin 2 | Angpt2 | Yes | No | No |
| 31 | AA818262 | angiopoietin-like 4 | Angptl4 | Yes | Yes | Yes |
| 32 | AF201331 | angiotensin I converting enzyme (peptidyl-dipeptidase A) 1 | Ace | Yes | No | No |
| 33 | BF552873 | angiotensin II receptor, type 2 | Agtr2 | No | N/A | N/A |
| 34 | NM_031009 | angiotensin receptor 1b | Agtr1b | Yes | No | No |
| 35 | NM_134432 | angiotensinogen (serpin peptidase inhibitor, clade A, member 8) | Agt | No | N/A | N/A |
| 36 | AJ428573 | ankyrin 3, epithelial | Ank3 | Yes | No | No |
| 37 | L81174 | ankyrin repeat domain 1 (cardiac muscle) | Ankrd1 | Yes | Yes | No |
| 38 | NM_012904 | annexin A1 | Anxa1 | Yes | No | No |
| 39 | NM_024155 | annexin A4 | Anxa4 | Yes | No | No |
| 40 | NM_013132 | annexin A5 | Anxa5 | Yes | No | No |
| 41 | BI275921 | anterior pharynx defective 1a homolog (C. elegans) | Aph1a | Yes | No | No |
| 42 | NM_133400 | apobec-1 complementation factor | Acf | Yes | No | No |
| 43 | J02582 | apolipoprotein E | Apoe | Yes | No | Yes |
| 44 | NM_053720 | apoptosis antagonizing transcription factor | Aatf | Yes | No | No |
| **#** | **Accession #** | **NAME** | **Symbol** | **Present** | **Exp. 1** | **Exp. 2** |
| 45 | AI233249 | apoptosis inhibitor 5 (predicted) | Api5_predicted | Yes | No | No |
| 46 | AW144082 | Apoptosis, caspase activation inhibitor (predicted) | Aven_predicted | No | N/A | N/A |
| 47 | AA894233 | apoptosis-inducing factor (AIF)-like mitochondrion-associated inducer of death (predicted) | Amid_predicted | No | N/A | N/A |
| 48 | BE116857 | apoptotic chromatin condensation inducer 1 | Acin1 | Yes | No | Yes |
| 49 | AF218388 | apoptotic peptidase activating factor 1 | Apaf1 | Yes | No | No |
| 50 | L07268 | aquaporin 1 | Aqp1 | Yes | Yes | Yes |
| 51 | NM_019158 | aquaporin 8 | Aqp8 | Yes | No | Yes |
| 52 | NM_031010 | arachidonate 15-lipoxygenase | Alox15 | Yes | No | No |
| 53 | BF285345 | arrestin, beta 2 | Arrb2 | No | N/A | N/A |
| 54 | NM_013149 | aryl hydrocarbon receptor | Ahr | Yes | No | No |
| 55 | NM_012780 | aryl hydrocarbon receptor nuclear translocator | Arnt | Yes | No | Yes |
| 56 | NM_021590 | aryl hydrocarbon receptor-interacting protein-like 1 | Aipl1 | Yes | No | No |
| 57 | BI274345 | ataxin 10 | Atxn10 | Yes | Yes | Yes |
| 58 | NM_058213 | ATPase, Ca++ transporting, cardiac muscle, fast twitch 1 | Atp2a1 | No | N/A | N/A |
| 59 | J04024 | ATPase, Ca++ transporting, cardiac muscle, slow twitch 2 | Atp2a2 | Yes | No | Yes |
| 60 | AY082609 | ATP-binding cassette, sub-family B (MDR/TAP), member 1 /// ATP-binding cassette, sub-family B (MDR/TAP), member 1A | Abcb1 /// Abcb1a | Yes | No | No |
| 61 | NM_017228 | atrophin 1 | Atn1 | Yes | No | No |
| 62 | AI169001 | autophagy-related 12 (yeast) | Atg12 | Yes | No | No |
| 63 | AI406520 | AXL receptor tyrosine kinase | Axl | Yes | No | No |
| 64 | NM_021752 | baculoviral IAP repeat-containing 2 | Birc2 | Yes | Yes | Yes |
| 65 | NM_023987 | baculoviral IAP repeat-containing 3 | Birc3 | Yes | Yes | Yes |
| 66 | AF304333 | baculoviral IAP repeat-containing 4 | Birc4 | Yes | No | Yes |
| 67 | NM_022274 | baculoviral IAP repeat-containing 5 | Birc5 | Yes | No | No |
| 68 | NM_031328 | B-cell CLL/lymphoma 10 | Bcl10 | Yes | Yes | Yes |
| 69 | NM_016993 | B-cell leukemia/lymphoma 2 | Bcl2 | Yes | No | Yes |
| 70 | NM_133416 | B-cell leukemia/lymphoma 2 related protein A1 | Bcl2a1 | Yes | No | Yes |
| 71 | AI172204 | B-cell receptor-associated protein 29 | Bcap29 | Yes | No | Yes |
| 72 | AI409930 | B-cell receptor-associated protein 31 | Bcap31 | Yes | No | No |
| 73 | NM_017258 | B-cell translocation gene 1, anti-proliferative | Btg1 | Yes | Yes | Yes |
| 74 | BI288701 | B-cell translocation gene 2, anti-proliferative | Btg2 | Yes | Yes | Yes |
| 75 | NM_139258 | Bcl2 modifying factor | Bmf | No | N/A | N/A |
| 76 | NM_053420 | BCL2/adenovirus E1B 19 kDa-interacting protein 3 | Bnip3 | Yes | No | Yes |
| 77 | NM_080888 | BCL2/adenovirus E1B 19 kDa-interacting protein 3-like | Bnip3l | Yes | No | Yes |
| 78 | NM_080897 | BCL2/adenovirus E1B 19kDa-interacting protein 1 | Bnip1 | Yes | No | No |
| 79 | AI178277 | BCL2/adenovirus E1B 19kDa-interacting protein 1, NIP2 (predicted) | Bnip2_predicted | Yes | No | No |
| 80 | NM_053812 | BCL2-antagonist/killer 1 | Bak1 | Yes | No | No |
| 81 | BI280304 | Bcl2-associated athanogene 1 (predicted) | Bag1_predicted | Yes | Yes | No |
| 82 | AI231792 | Bcl2-associated athanogene 3 | Bag3 | Yes | Yes | No |
| 83 | BI282898 | BCL2-associated athanogene 5 | Bag5 | Yes | No | Yes |
| 84 | AF279911 | bcl2-associated death promoter | Bad | Yes | No | No |
| 85 | AI717547 | BCL2-associated transcription factor 1 | Bclaf1 | Yes | No | Yes |
| 86 | AF235993 | Bcl2-associated X protein | Bax | Yes | No | Yes |
| 87 | U72350 | Bcl2-like 1 | Bcl2l1 | Yes | No | Yes |
| 88 | NM_053733 | Bcl2-like 10 | Bcl2l10 | Yes | No | No |
| 89 | NM_022612 | BCL2-like 11 (apoptosis facilitator) | Bcl2l11 | Yes | Yes | No |
| 90 | AI227978 | BCL2-like 12 (proline rich) (predicted) | Bcl2l12_predicted | No | N/A | N/A |
| **#** | **Accession #** | **NAME** | **Symbol** | **Present** | **Exp. 1** | **Exp. 2** |
| 91 | AA892271 | BCL2-like 13 (apoptosis facilitator) (predicted) | Bcl2l13_predicted | Yes | No | Yes |
| 92 | NM_021850 | Bcl2-like 2 | Bcl2l2 | No | N/A | N/A |
| 93 | AF051093 | Bcl-2-related ovarian killer protein | Bok | Yes | Yes | No |
| 94 | NM_053739 | beclin 1 (coiled-coil, myosin-like BCL2-interacting protein) | Becn1 | Yes | No | No |
| 95 | AI008680 | benzodiazepine receptor, peripheral | Bzrp | Yes | No | Yes |
| 96 | NM_057130 | BH3 interacting (with BCL2 family) domain, apoptosis agonist | Bid3 | No | N/A | N/A |
| 97 | AF136282 | BH3 interacting domain death agonist | Bid | No | N/A | N/A |
| 98 | AI177631 | bifunctional apoptosis regulator | Bfar | Yes | No | No |
| 99 | NM_012827 | bone morphogenetic protein 4 | Bmp4 | Yes | No | No |
| 100 | BE118651 | bone morphogenic protein receptor, type II (serine/threonine kinase) | Bmpr2 | Yes | No | No |
| 101 | AA851481 | brain and reproductive organ-expressed protein | Bre | Yes | No | No |
| 102 | X67108 | brain derived neurotrophic factor | Bdnf | Yes | Yes | No |
| 103 | AI169085 | brain zinc finger protein | Zfp179 | Yes | No | No |
| 104 | NM_017253 | branched chain aminotransferase 1, cytosolic | Bcat1 | Yes | No | No |
| 105 | NM_022622 | BRCA1 associated RING domain 1 | Bard1 | No | N/A | N/A |
| 106 | BF404972 | Breast cancer 1 | Brca1 | Yes | No | No |
| 107 | NM_012931 | breast cancer anti-estrogen resistance 1 | Bcar1 | Yes | Yes | Yes |
| 108 | NM_134413 | BTB (POZ) domain containing 14B | Btbd14b | No | N/A | N/A |
| 109 | NM_031334 | cadherin 1 | Cdh1 | No | N/A | N/A |
| 110 | NM_019161 | cadherin 22 | Cdh2 | Yes | No | No |
| 111 | AF061947 | calcineurin binding protein 1 | Cdh22 | Yes | No | No |
| 112 | BM958511 | calcium binding protein p22 | Cabin1 | Yes | No | Yes |
| 113 | AB070350 | calcium binding protein p22 /// similar to calcium binding protein P22 (predicted) /// similar to calcium binding protein P22 | Chp | Yes | No | No |
| 114 | BF404381 | Calcium/calmodulin-dependent protein kinase II, alpha | RGD1565588_predict | Yes | No | No |
| 115 | NM_016996 | calcium-sensing receptor | Casr | No | N/A | N/A |
| 116 | NM_019152 | calpain 1 | Capn1 | Yes | No | No |
| 117 | NM_053295 | calpastatin | Cast | Yes | No | No |
| 118 | NM_022399 | calreticulin | Calr | Yes | Yes | Yes |
| 119 | NM_032462 | calsenilin, presenilin binding protein, EF hand transcription factor | Csen | No | N/A | N/A |
| 120 | NM_031017 | cAMP responsive element binding protein 1 | Creb1 | No | N/A | N/A |
| 121 | NM_017334 | cAMP responsive element modulator | Crem | Yes | Yes | Yes |
| 122 | NM_012784 | cannabinoid receptor 1 (brain) | Cnr1 | No | N/A | N/A |
| 123 | AW252112 | carbonic anhydrase 11 | Car11 | Yes | No | Yes |
| 124 | BF281311 | casein kinase 2, beta subunit | Csnk2b | Yes | Yes | No |
| 125 | NM_057138 | CASP8 and FADD-like apoptosis regulator | Cflar | Yes | No | Yes |
| 126 | D85899 | caspase 1 | Casp1 | Yes | No | Yes |
| 127 | NM_130422 | caspase 12 | Casp12 | Yes | No | Yes |
| 128 | AF136231 | caspase 2 | Casp2 | Yes | Yes | No |
| 129 | BM387008 | caspase 3, apoptosis related cysteine protease | Casp3 | Yes | No | No |
| 130 | NM_053736 | caspase 4, apoptosis-related cysteine peptidase | Casp4 | Yes | No | Yes |
| 131 | NM_031775 | caspase 6 | Casp6 | No | N/A | N/A |
| 132 | BF283754 | caspase 7 | Casp7 | Yes | Yes | Yes |
| 133 | BF282281 | caspase 8 associated protein 2 (predicted) | Casp8ap2_predicted | Yes | No | Yes |
| 134 | AF262319 | caspase 9 | Casp9 | Yes | No | No |
| 135 | NM_022303 | caspase recruitment domain family, member 9 | Card9 | Yes | No | No |
| 136 | AI136555 | castration induced prostatic apoptosis-related protein 1 | Cipar1 | Yes | No | No |
| **#** | **Accession #** | **NAME** | **Symbol** | **Present** | **Exp. 1** | **Exp. 2** |
| 137 | NM_022597 | cathepsin B | Ctsb | Yes | No | No |
| 138 | NM_134334 | cathepsin D | Ctsd | Yes | No | Yes |
| 139 | AI548979 | cationic trypsinogen | LOC286911 | No | N/A | N/A |
| 140 | NM_024125 | CCAAT/enhancer binding protein (C/EBP), beta | Cebpb | Yes | Yes | Yes |
| 141 | NM_021744 | CD14 antigen | Cd14 | Yes | Yes | Yes |
| 142 | NM_017079 | CD1d1 antigen | Cd1d1 | Yes | No | No |
| 143 | NM_012830 | CD2 antigen | Cd2 | Yes | No | Yes |
| 144 | NM_013121 | CD28 antigen | Cd28 | No | N/A | N/A |
| 145 | AI044631 | CD3 antigen, gamma polypeptide | Cd3g_predicted | Yes | No | Yes |
| 146 | D30795 | CD38 antigen | Cd38 | Yes | No | No |
| 147 | AF065147 | CD44 antigen | Cd44 | Yes | No | No |
| 148 | NM_019295 | CD5 antigen | Cd5 | Yes | No | No |
| 149 | NM_012523 | CD53 antigen | Cd53 | Yes | No | No |
| 150 | NM_013069 | CD74 antigen (invariant polypeptide of major histocompatibility complex, class II antigen-associated) | Cd74 | Yes | No | No |
| 151 | NM_031755 | CEA-related cell adhesion molecule 1 | Ceacam1 | Yes | No | No |
| 152 | U23056 | CEA-related cell adhesion molecule 1 /// CEA-related cell adhesion molecule 10 | Ceacam1 | No | N/A | N/A |
| 153 | BF284899 | cell death-inducing DNA fragmentation factor, alpha subunit-like effector A (predicted) | Cidea_predicted | Yes | No | No |
| 154 | L24388 | cell division cycle 2 homolog (S.pombe)-like 1 | Cdc2 | No | N/A | N/A |
| 155 | AI059933 | Cell division cycle 25 homolog A (S. cerevisiae) | Cdc2 | Yes | No | No |
| 156 | NM_023026 | centaurin, gamma 1 | Ceng1a | No | N/A | N/A |
| 157 | NM_031530 | chemokine (C-C motif) ligand 2 | Ccl2 | Yes | Yes | Yes |
| 158 | NM_031116 | chemokine (C-C motif) ligand 5 | Ccl5 | Yes | No | Yes |
| 159 | BE095824 | chemokine (C-C motif) ligand 6 | Ccl6 | Yes | No | No |
| 160 | AA945737 | chemokine (C-X-C motif) receptor 4 | Cxcr4 | Yes | No | Yes |
| 161 | AI012221 | chloride intracellular channel 1 | Clic1 | Yes | Yes | Yes |
| 162 | NM_012829 | cholecystokinin | Cck | Yes | No | No |
| 163 | NM_012832 | cholinergic receptor, nicotinic, alpha polypeptide 7 | Chrna7 | No | N/A | N/A |
| 164 | AI171615 | chromosome segregation 1-like (S. cerevisiae) (predicted) | Cse1l_predicted | Yes | No | No |
| 165 | NM_013092 | chymase 1, mast cell | Cma1 | No | N/A | N/A |
| 166 | AA957183 | Citron | Cit | Yes | No | No |
| 167 | BG673439 | claudin 11 | Cldn11 | Yes | No | Yes |
| 168 | AF314657 | clusterin | Clu | Yes | No | Yes |
| 169 | NM_012950 | coagulation factor II (thrombin) receptor | F2r | Yes | Yes | Yes |
| 170 | NM_013057 | coagulation factor III | F3 | Yes | Yes | No |
| 171 | BM389673 | cofilin 1, non-muscle | Cfl1 | Yes | No | Yes |
| 172 | AF092207 | coiled-coil domain containing 5 | Ccdc5 | Yes | No | Yes |
| 173 | U00620 | colony stimulating factor 2 (granulocyte-macrophage) | Csf2 | Yes | No | No |
| 174 | NM_130825 | comparative gene identification transcript 94 | Cgi94 | No | N/A | N/A |
| 175 | NM_032060 | complement component 3a receptor 1 | C3ar1 | Yes | No | No |
| 176 | NM_053619 | complement component 5, receptor 1 | C5r1 | Yes | No | No |
| 177 | AA819870 | complement component 8, beta polypeptide (mapped) | C8b | Yes | No | No |
| 178 | NM_057146 | complement component 9 | C9 | No | N/A | N/A |
| 179 | AW916366 | COP9 (constitutive photomorphogenic) homolog, subunit 3 (Arabidopsis thaliana) | Cops3 | Yes | No | Yes |
| 180 | NM_031019 | corticotropin releasing hormone | Crh | No | N/A | N/A |
| 181 | AW433973 | craniofacial development protein 1 | Cfdp1 | Yes | Yes | No |
| 182 | U47922 | crystallin, alpha A | Cryaa | No | N/A | N/A |
| **#** | **Accession #** | **NAME** | **Symbol** | **Present** | **Exp. 1** | **Exp. 2** |
| 183 | NM_012935 | crystallin, alpha B | Cryab | Yes | No | No |
| 184 | AF090695 | CUG triplet repeat, RNA binding protein 2 | Cugbp2 | Yes | Yes | Yes |
| 185 | BI284428 | cullin 1 (predicted) | Cul1_predicted | Yes | Yes | No |
| 186 | BI295890 | cullin 2 (predicted) | Cul2_predicted | Yes | No | No |
| 187 | BI285751 | cullin 3 (predicted) | Cul3_predicted | Yes | No | Yes |
| 188 | NM_022683 | cullin 5 | Cul5 | Yes | No | No |
| 189 | X64589 | cyclin B1 | Ccnb1 | Yes | No | No |
| 190 | AW913890 | cyclin E | Ccne | No | N/A | N/A |
| 191 | NM_080885 | cyclin-dependent kinase 5 | Cdk5 | Yes | No | No |
| 192 | NM_053891 | cyclin-dependent kinase 5, regulatory subunit 1 (p35) | Cdk5r1 | No | N/A | N/A |
| 193 | H31766 | cyclin-dependent kinase 9 (CDC2-related kinase) | Cdk9 | Yes | No | Yes |
| 194 | AI010427 | cyclin-dependent kinase inhibitor 1A | Cdkn1a | Yes | Yes | Yes |
| 195 | AI013919 | cyclin-dependent kinase inhibitor 1C (P57) | Cdkn1c | Yes | No | Yes |
| 196 | AF474976 | cyclin-dependent kinase inhibitor 2A | Cdkn2a | No | N/A | N/A |
| 197 | AI409867 | cystatin B | Cstb | Yes | No | Yes |
| 198 | BG666933 | cystatin C | Cst3 | Yes | No | No |
| 199 | NM_031327 | cysteine rich protein 61 | Cyr61 | Yes | Yes | No |
| 200 | NM_023965 | cytochrome b-245, beta polypeptide | Cybb | Yes | No | No |
| 201 | NM_012839 | cytochrome c, somatic | Cycs | Yes | Yes | Yes |
| 202 | NM_012840 | cytochrome c, testis | Cyct | No | N/A | N/A |
| 203 | NM_012840 | cytochrome c, testis /// phosphodiesterase 11A | Cyctpd11 | No | N/A | N/A |
| 204 | X00469 | cytochrome P450, family 1, subfamily a, polypeptide 1 | Cyp27b1 | No | N/A | N/A |
| 205 | NM_031543 | cytochrome P450, family 2, subfamily e, polypeptide 1 | Cyp1e1 | Yes | No | No |
| 206 | BF285068 | cytokine induced apoptosis inhibitor 1 | Ciapin1 | Yes | No | No |
| 207 | BI298817 | Cytotoxic granule-associated RNA binding protein 1 | Tia1 | No | N/A | N/A |
| 208 | AI169146 | D4, zinc and double PHD fingers family 2 (predicted) | Dpf2_predicted | Yes | No | No |
| 209 | AI408110 | DEAD (Asp-Glu-Ala-Asp) box polypeptide 19 | Ddx19 | Yes | No | No |
| 210 | BM389310 | DEAD (Asp-Glu-Ala-Asp) box polypeptide 41 (predicted) | Ddx41_predicted | Yes | No | Yes |
| 211 | BI285645 | death associated protein 3 | Dap3 | Yes | No | No |
| 212 | AA818353 | death associated protein kinase 1 (predicted) | DapK1_predicted | Yes | No | Yes |
| 213 | NM_031800 | death effector domain-containing | Dedd | No | N/A | N/A |
| 214 | NM_022526 | death-associated protein | Dap | No | N/A | N/A |
| 215 | NM_022546 | death-associated protein kinase 3 | Dapk3 | Yes | No | Yes |
| 216 | AI013627 | defender against cell death 1 | Dad1 | Yes | No | No |
| 217 | NM_080482 | deleted in bladder cancer chromosome region candidate 1 (human) | Dbccr | Yes | No | No |
| 218 | NM_012841 | deleted in colorectal carcinoma | Dcc | Yes | No | No |
| 219 | NM_013097 | deoxyribonuclease I | Dnase1 | No | N/A | N/A |
| 220 | AF178975 | deoxyribonuclease II | Dnase2 | Yes | No | No |
| 221 | NM_053907 | deoxyribonuclease I-like 3 | LOC681124 | Yes | No | No |
| 222 | NM_022531 | desmin | Des | Yes | No | No |
| 223 | BE110572 | diablo homolog (Drosophila) | Diablo | Yes | No | No |
| 224 | AI236726 | DNA fragmentation factor, alpha subunit | Dffa | Yes | No | Yes |
| 225 | NM_053362 | DNA fragmentation factor, beta subunit | Dffb | Yes | No | No |
| 226 | NM_024134 | DNA-damage inducible transcript 3 | Ddit3 | Yes | Yes | Yes |
| 227 | NM_080906 | DNA-damage-inducible transcript 4 | Ddit4 | Yes | Yes | Yes |
| 228 | BI282224 | DnaJ (Hsp40) homolog, subfamily A, member 3 | LOC294513 | Yes | No | Yes |
| **#** | **Accession #** | **NAME** | **Symbol** | **Present** | **Exp. 1** | **Exp. 2** |
| 229 | BM384926 | DnaJ (Hsp40) homolog, subfamily B, member 1 (predicted) | Dnajb1_predicted | Yes | Yes | Yes |
| 230 | NM_012699 | DnaJ (Hsp40) homolog, subfamily B, member 9 | Dnajb9 | Yes | No | No |
| 231 | BI285682 | DnaJ (Hsp40) homolog, subfamily C, member 7 | Dnajc7 | Yes | Yes | No |
| 232 | BF406540 | DnaJ (Hsp40) related, subfamily B, member 13 | Dnajb13 | No | N/A | N/A |
| 233 | L12407 | dopamine beta hydroxylase | Dbh | Yes | No | Yes |
| 234 | NM_012547 | dopamine receptor 2 | Drd2 | No | N/A | N/A |
| 235 | M35077 | dopamine receptor D1A | Drd1a | No | N/A | N/A |
| 236 | BE110108 | dual specificity phosphatase 1 | Dusp1 | Yes | No | Yes |
| 237 | AI172067 | dual specificity phosphatase 22 (predicted) | Dusp22_predicted | Yes | No | Yes |
| 238 | U23438 | dual specificity phosphatase 4 | Dusp4 | Yes | No | No |
| 239 | NM_133578 | dual specificity phosphatase 5 | Dusp5 | Yes | Yes | Yes |
| 240 | NM_053883 | dual specificity phosphatase 6 | Dusp6 | Yes | Yes | Yes |
| 241 | L24562 | dynamin 2 | Dnm3 | No | N/A | N/A |
| 242 | NM_053319 | dynein light chain LC8-type 1 | Dynll1 | Yes | Yes | No |
| 243 | NM_012551 | early growth response 1 | Egr1 | Yes | Yes | Yes |
| 244 | AF115249 | endothelial differentiation, sphingolipid G-protein-coupled receptor, 8 | Edg8 | No | N/A | N/A |
| 245 | NM_023090 | endothelial PAS domain protein 1 | Epas1 | Yes | No | Yes |
| 246 | NM_053596 | endothelin converting enzyme 1 | Ece1 | Yes | No | Yes |
| 247 | AB023896 | endothelin converting enzyme-like 1 | Ecel1 | No | N/A | N/A |
| 248 | X57764 | endothelin receptor type B | Ednrb | Yes | Yes | Yes |
| 249 | BI291645 | engulfment and cell motility 3, ced-12 homolog (C. elegans) | Elmo3 | No | N/A | N/A |
| 250 | NM_012842 | epidermal growth factor | Egfr | No | N/A | N/A |
| 251 | M37394 | epidermal growth factor receptor | Egfr | Yes | No | No |
| 252 | AF187818 | epidermal growth factor receptor /// peptidase D (mapped) | Egfr /// Pepd_mapped | Yes | No | No |
| 253 | BF564277 | epilepsy, progressive myoclonic epilepsy, type 2 gene alpha | Epme | No | N/A | N/A |
| 254 | NM_017001 | erythropoietin | Epo | No | N/A | N/A |
| 255 | AA866269 | Estrogen receptor 1 | Esr1 | No | N/A | N/A |
| 256 | AF042058 | estrogen receptor 2 beta | Esr2b | No | N/A | N/A |
| 257 | BF398331 | estrogen receptor-binding fragment-associated gene 9 | Ebag9 | Yes | Yes | Yes |
| 258 | AI412114 | etoposide induced 2.4 mRNA | Ei24 | Yes | No | No |
| 259 | NM_012660 | eukaryotic translation elongation factor 1 alpha 2 | Eef1a2 | Yes | No | No |
| 260 | AI600237 | eukaryotic translation elongation factor 1 epsilon 1 (predicted) | Eef1e1_predicted | Yes | No | Yes |
| 261 | NM_053950 | eukaryotic translation initiation factor 2B, subunit 4 delta | Eif2b4 | Yes | No | No |
| 262 | NM_053974 | eukaryotic translation initiation factor 4E | Eif4e | Yes | No | No |
| 263 | BI283681 | eukaryotic translation initiation factor 5A | Eif5a | Yes | No | Yes |
| 264 | BM388758 | excision repair cross-complementing rodent repair deficiency, complementation group 3 | Ercc3 | Yes | No | No |
| 265 | D13374 | expressed in non-metastatic cells 1 | Nme1 | Yes | No | No |
| 266 | AI385371 | extra spindle poles like 1 (S. cerevisiae) (predicted) | Espl1_predicted | No | N/A | N/A |
| 267 | NM_080895 | Fas apoptotic inhibitory molecule | Faim | Yes | Yes | Yes |
| 268 | AF044201 | Fas apoptotic inhibitory molecule 2 | Faim2 | No | N/A | N/A |
| 269 | NM_080891 | Fas death domain-associated protein | Daxx | No | N/A | N/A |
| 270 | NM_012908 | Fas ligand (TNF superfamily, member 6) | Faslg | Yes | No | No |
| 271 | AI227743 | Fas-activated serine/threonine kinase | Fastk | Yes | No | No |
| 272 | NM_130406 | Fas-associated factor 1 | Faf1 | Yes | No | Yes |
| 273 | NM_053843 | Fc receptor, IgG, low affinity III /// Fc gamma receptor II beta | Fcgr3 /// LOC498276 | Yes | Yes | Yes |
| 274 | AA999104 | Feminization 1 homolog b (C. elegans) (predicted) | Fem1_predicted | Yes | No | No |
| **#** | **Accession #** | **NAME** | **Symbol** | **Present** | **Exp. 1** | **Exp. 2** |
| 275 | NM_019305 | fibroblast growth factor 2 | Fgf2 | Yes | No | No |
| 276 | NM_130817 | fibroblast growth factor 3 | Fgf3 | No | N/A | N/A |
| 277 | AB079673 | fibroblast growth factor 4 | Fgf4 | No | N/A | N/A |
| 278 | NM_133286 | fibroblast growth factor 8 | Fgf8 | No | N/A | N/A |
| 279 | S54008 | Fibroblast growth factor receptor 1 | Fgfr1 | Yes | No | No |
| 280 | NM_053429 | fibroblast growth factor receptor 3 | Fgfr3 | No | N/A | N/A |
| 281 | AA893484 | fibronectin 1 | Fn1 | Yes | No | No |
| 282 | AI103600 | Filamin C, gamma (actin binding protein 280) (predicted) | Flnc_predicted | Yes | Yes | No |
| 283 | AF040256 | folate hydrolase | Folh1 | No | N/A | N/A |
| 284 | M36804 | follicle stimulating hormone beta | Fshb | Yes | No | No |
| 285 | NM_012561 | follistatin | Fst | No | N/A | N/A |
| 286 | BI295511 | forkhead box O1A | Foxo1a | Yes | No | No |
| 287 | AI231684 | forkhead box O3a (predicted) | Foxo3a_predicted | No | N/A | N/A |
| 288 | NM_012953 | fos-like antigen 1 | Fosl1 | Yes | No | No |
| 289 | NM_012954 | fos-like antigen 2 /// FBJ osteosarcoma oncogene B | Fosl2 /// Fosb | Yes | Yes | Yes |
| 290 | NM_017181 | fumarylacetoacetate hydrolase | Fah | Yes | No | No |
| 291 | BE108192 | G1 to S phase transition 1 | Gspt1 | Yes | Yes | Yes |
| 292 | NM_033237 | galanin | Gal | No | N/A | N/A |
| 293 | NM_019172 | galanin receptor 2 | Galr2 | No | N/A | N/A |
| 294 | NM_053840 | gamma-glutamyltransferase 1 | Ggt1 | No | N/A | N/A |
| 295 | NM_019281 | gap junction membrane channel protein alpha 9 | Gja9 | No | N/A | N/A |
| 296 | NM_053388 | gap junction membrane channel protein beta 6 | Gja6 | No | N/A | N/A |
| 297 | NM_012849 | gastrin | Gast | No | N/A | N/A |
| 298 | AA945758 | gb:AA945758 /DB_XREF=gi:3105674 /DB_XREF=EST201257 /CLONE=RLUAS87 /FEA=EST /CNT=13 /TID=Rn.7908.1 /TIER=Stack /STK=8 /UG=Rn.7908 /UG_TITLE=ESTs | NS | No | N/A | N/A |
| 299 | AI230220 | gb:AI230220 /DB_XREF=gi:3814107 /DB_XREF=EST226915 /CLONE=REMCT79 /FEA=EST /CNT=9 /TID=Rn.24381.1 /TIER=Stack /STK=7 /UG=Rn.24381 /UG_TITLE=ESTs, Moderately similar to MLE3 RAT MYOSIN LIGHT CHAIN 3, SKELETAL MUSCLE ISOFORM (R.norvegicus) | NS | No | N/A | N/A |
| 300 | BF555051 | gb:BF555051 /DB_XREF=gi:11664781 /DB_XREF=UI-R-E0-cg-f-04-0-UI.r1 /CLONE=UI-R-E0-cg-f-04-0-UI /FEA=EST /CNT=3 /TID=Rn.65517.1 /TIER=ConsEnd /STK=1 /UG=Rn.65517 /UG_TITLE=ESTs, Weakly similar to VITAMIN K-DEPENDENT PROTEIN S PRECURSOR (R.norvegicus) | NS | No | N/A | N/A |
| 301 | BM384229 | gb:BM384229 /DB_XREF=gi:18184282 /DB_XREF=UI-R-DZ0-cks-c-03-0-UI.s1 /CLONE=UI-R-DZ0-cks-c-03-0-UI /FEA=EST /CNT=12 /TID=Rn.14615.1 /TIER=Stack /STK=11 /UG=Rn.14615 /UG_TITLE=ESTs, Highly similar to TRA2 MOUSE TNF RECEPTOR ASSOCIATED FACTOR 2 (M.musculus) | NS | No | N/A | N/A |
| 302 | J02582 | gb:J02582 /DB_XREF=gi:202957 /FEA=DNA_2 /CNT=1 /TID=Rn.64667.1 /TIER=ConsEnd /STK=0 /UG=Rn.64667 /UG_TITLE=Rat apolipoprotein E gene, complete cds /DEF=Rat apolipoprotein E gene, complete cds | NS | No | N/A | N/A |
| 303 | BI285576 | gelsolin | Gsn | Yes | Yes | No |
| 304 | NM_021669 | ghrelin precursor | Ghrl | No | N/A | N/A |
| 305 | NM_019139 | glial cell line derived neurotrophic factor | Gdnf | No | N/A | N/A |
| 306 | BF281741 | glioma tumor suppressor candidate region gene 2 | Gltscr2 | Yes | No | No |
| 307 | NM_012728 | glucagon-like peptide 1 receptor | Glpr1r | No | N/A | N/A |
| 308 | BI283882 | glucose phosphate isomerase | Gpi | Yes | No | No |
| 309 | NM_017006 | glucose-6-phosphate dehydrogenase X-linked | G6pdx | Yes | Yes | Yes |
| 310 | U08259 | glutamate receptor, ionotropic, NMDA2C | Grin2c | Yes | No | No |
| 311 | NM_017010 | glutamate receptor, ionotropic, N-methyl D-aspartate 1 | Grin1 | No | N/A | N/A |
| 312 | AF001423 | glutamate receptor, ionotropic, N-methyl D-aspartate 2A | Grin2a | Yes | No | Yes |
| 313 | M91562 | glutamate receptor, ionotropic, N-methyl D-aspartate 2B | Grin2b | Yes | No | No |
| 314 | NM_017011 | glutamate receptor, metabotropic 1 | Gria2bm1 | Yes | No | Yes |
| 315 | M92075 | glutamate receptor, metabotropic 2 | Grm2 | No | N/A | N/A |
| **#** | **Accession #** | **NAME** | **Symbol** | **Present** | **Exp. 1** | **Exp. 2** |
| 316 | AW522430 | glutamate receptor, metabotropic 3 | Grm3 | No | N/A | N/A |
| 317 | NM_022202 | glutamate receptor, metabotropic 8 | Gria2bm8 | Yes | No | No |
| 318 | J05181 | glutamate-cysteine ligase, catalytic subunit | Gclc | Yes | No | Yes |
| 319 | BG380882 | glutaminyl-tRNA synthetase /// similar to glutaminyl-tRNA synthetase (predicted) | Qars_predicted | Yes | No | No |
| 320 | NM_022278 | glutaredoxin 1 (thioltransferase) | Glrx1 | Yes | No | No |
| 321 | S41066 | glutathione peroxidase 1 | Gpx1 | Yes | No | Yes |
| 322 | NM_017165 | glutathione peroxidase 4 | Gpx4 | Yes | Yes | No |
| 323 | NM_017013 | glutathione-S-transferase, alpha type2 | Gsta2 | Yes | No | No |
| 324 | X02904 | glutathione-S-transferase, pi 1 /// glutathione S-transferase, pi 2 | Gstp1 /// Gstp2 | Yes | Yes | No |
| 325 | NM_017008 | glyceraldehyde-3-phosphate dehydrogenase /// similar to glyceraldehyde-3-phosphate dehydrogenase (predicted) /// similar to glyceraldehyde-3-phosphate dehydrogenase (predicted) /// similar to glyceraldehyde-3-phosphate dehydrogenase (predicted) /// similar to glyceraldehyde-3-phosphate dehydrogenase (predicted) | Gapdh /// RGD1564688_predicted /// RGD1564351_predicted /// RGD1561683_predicted /// RGD1565368_predicted | Yes | No | No |
| 326 | BF287444 | glycogen synthase kinase 3 beta | Gsk3b | Yes | No | Yes |
| 327 | AI103970 | glyoxylase 1 | Glo1 | Yes | No | Yes |
| 328 | BM391371 | goliath | LOC652955 | Yes | Yes | No |
| 329 | NM_031038 | gonadotropin releasing hormone receptor | Gnrhr | Yes | No | No |
| 330 | NM_012767 | gonadotropin-releasing hormone 1 | Gnrh1 | Yes | No | No |
| 331 | M34097 | granzyme B | Gzmb | Yes | No | Yes |
| 332 | U57063 | granzyme G | Gzmg | Yes | No | No |
| 333 | NM_019282 | gremlin 1 homolog, cysteine knot superfamily (Xenopus laevis) | Grem1 | No | N/A | N/A |
| 334 | NM_024127 | growth arrest and DNA-damage-inducible 45 alpha | Gadd45a | Yes | Yes | Yes |
| 335 | BI287978 | growth arrest and DNA-damage-inducible 45 beta | Gadd45b | Yes | Yes | Yes |
| 336 | AI599423 | growth arrest and DNA-damage-inducible 45 gamma | Gadd45g | Yes | Yes | Yes |
| 337 | NM_057100 | growth arrest specific 6 | Gas6 | Yes | Yes | Yes |
| 338 | X62853 | growth factor receptor bound protein 2 | Grb2 | Yes | No | No |
| 339 | V01238 | growth hormone 1 | Gh1 | No | N/A | N/A |
| 340 | AI170771 | growth hormone receptor | Ghr | Yes | No | No |
| 341 | U94321 | growth hormone secretagogue receptor | Gshr | Yes | No | No |
| 342 | NM_024356 | GTP cyclohydrolase 1 | Gch | Yes | Yes | Yes |
| 343 | BM389208 | GTPase, IMAP family member 4 | Gimap4 | Yes | Yes | Yes |
| 344 | M12672 | guanine nucleotide binding protein, alpha inhibiting 2 | Gnai2 | Yes | Yes | No |
| 345 | BE117491 | Guanine nucleotide binding protein, alpha q polypeptide | Gnaq | Yes | No | No |
| 346 | BM390519 | GULP, engulfment adaptor PTB domain containing 1 | Gulp1 | Yes | Yes | Yes |
| 347 | BG379941 | Harvey rat sarcoma viral (v-Ha-ras) oncogene homolog | Hras | Yes | No | No |
| 348 | NM_012966 | heat shock 10 kDa protein 1 (chaperonin 10) | Hspe1 | Yes | No | Yes |
| 349 | AI236601 | heat shock 105kDa/110kDa protein 1 | Hsph1 | Yes | Yes | No |
| 350 | NM_031970 | heat shock 27kDa protein 1 | Hspb1 | Yes | Yes | Yes |
| 351 | NM_031971 | heat shock 70kD protein 1A /// heat shock 70kD protein 1B (mapped) | Hspa1a /// Hspa1b_mapped | Yes | Yes | No |
| 352 | BI278231 | heat shock 70kD protein 1B (mapped) | Hspa1b_mapped | Yes | Yes | No |
| 353 | M14050 | heat shock 70kDa protein 5 (glucose-regulated protein) | Hspa5 | Yes | Yes | Yes |
| 354 | BI282281 | heat shock 70kDa protein 9A (predicted) | Hspa9a_predicted | Yes | No | Yes |
| **#** | **Accession #** | **NAME** | **Symbol** | **Present** | **Exp. 1** | **Exp. 2** |
| 355 | AI237389 | heat shock 90kDa protein 1, beta | Hspcb | Yes | Yes | Yes |
| 356 | NM_022229 | heat shock protein 1 (chaperonin) | Hspd1 | Yes | No | Yes |
| 357 | BG671521 | heat shock protein 1, alpha | Hspca | Yes | Yes | No |
| 358 | AF077354 | heat shock protein 4 | Hspa4 | Yes | No | Yes |
| 359 | NM_012580 | heme oxygenase (decycling) 1 | Hmox1 | Yes | Yes | Yes |
| 360 | NM_013185 | hemopoietic cell kinase | Hck | Yes | No | Yes |
| 361 | NM_012734 | hexokinase 1 | Hk1 | Yes | No | No |
| 362 | NM_012735 | hexokinase 2 | Hk2 | Yes | Yes | Yes |
| 363 | BG378885 | high mobility group AT-hook 1 | Hmga1 | Yes | No | No |
| 364 | BE107162 | high mobility group box 1 | Hmgb1 | Yes | No | No |
| 365 | AF275734 | high mobility group box 1 /// similar to High mobility group protein 1 (HMG-1) (predicted) /// similar to Hmgb1 protein (predicted) /// similar to High mobility group protein 1 (HMG-1) (predicted) | Hmgb1 /// RGD1562312_predicted /// RGD1563786_predicted /// RGD1563012_predicted | Yes | No | No |
| 366 | AI180339 | histone deacetylase 1 (predicted) | Hdac1_predicted | Yes | No | No |
| 367 | NM_053448 | histone deacetylase 3 | Hdac3 | Yes | No | No |
| 368 | BF403027 | histone deacetylase 5 | Hdac5 | Yes | Yes | No |
| 369 | NM_053609 | HLA-B-associated transcript 3 | Bat3 | Yes | No | Yes |
| 370 | M37568 | homeo box C8 (mapped) | Hoxc8_mapped | No | N/A | N/A |
| 371 | BM392321 | homeodomain interacting protein kinase 2 (predicted) | Hipk2_predicted | Yes | No | Yes |
| 372 | NM_031787 | homeodomain interacting protein kinase 3 | Hipk3 | Yes | No | Yes |
| 373 | AB003726 | homer homolog 1 (Drosophila) | Homer1 | Yes | Yes | No |
| 374 | NM_053523 | homocysteine-inducible, endoplasmic reticulum stress-inducible, ubiquitin-like domain member 1 | Herpud1 | Yes | No | Yes |
| 375 | BE111733 | hormone-regulated proliferation associated protein 20 | Hrpap20 | Yes | No | No |
| 376 | AW253339 | Huntingtin interacting protein 1 | Hip1 | Yes | No | No |
| 377 | U18650 | Huntington disease gene homolog | Hdh | No | N/A | N/A |
| 378 | NM_019371 | hypothetical gene supported by NM_019371 | LOC497816 | Yes | No | Yes |
| 379 | BM390522 | hypothetical gene supported by NM_130426 | LOC497808 | No | N/A | N/A |
| 380 | H31665 | hypoxia induced gene 1 | Hig1 | Yes | No | No |
| 381 | NM_024359 | hypoxia inducible factor 1, alpha subunit | Hif1a | Yes | Yes | No |
| 382 | BI282904 | hypoxia up-regulated 1 | Hyou1 | Yes | No | Yes |
| 383 | AI176519 | immediate early response 3 | Ier3 | Yes | Yes | Yes |
| 384 | AI411947 | immunoglobulin heavy chain 1a (serum IgG2a) | Igh-1a | No | N/A | N/A |
| 385 | NM_023973 | indoleamine-pyrrole 2,3 dioxygenase | Indo | Yes | No | Yes |
| 386 | NM_012590 | inhibin alpha | Inha | Yes | No | No |
| 387 | NM_017128 | inhibin beta-A | Inhba | Yes | No | Yes |
| 388 | NM_013060 | inhibitor of DNA binding 2 | Id2 | Yes | No | Yes |
| 389 | AF000942 | inhibitor of DNA binding 3 | Id3 | Yes | Yes | Yes |
| 390 | NM_053355 | inhibitor of kappaB kinase beta | Ikbkb | Yes | No | No |
| 391 | J05510 | inositol 1,4,5-triphosphate receptor 1 | Itpr1 | Yes | No | No |
| 392 | NM_019311 | inositol polyphosphate-5-phosphatase D | Inppd5 | Yes | Yes | Yes |
| 393 | NM_019129 | insulin 1 | Igf2bp1 | Yes | No | No |
| 394 | NM_032074 | insulin receptor substrate 3 | Irs3 | Yes | Yes | Yes |
| 395 | M15481 | insulin-like growth factor 1 | Igf1 | Yes | No | Yes |
| 396 | NM_052807 | insulin-like growth factor 1 receptor | Igf1r | Yes | No | No |
| **#** | **Accession #** | **NAME** | **Symbol** | **Present** | **Exp. 1** | **Exp. 2** |
| 397 | NM_031511 | insulin-like growth factor 2 | Igf2 | Yes | No | No |
| 398 | NM_012588 | insulin-like growth factor binding protein 3 | Igfbp3 | Yes | Yes | No |
| 399 | BF282337 | integral membrane protein 2B | Itm2b | Yes | Yes | Yes |
| 400 | NM_017022 | integrin beta 1 (fibronectin receptor beta) | Itgb1 | Yes | Yes | Yes |
| 401 | NM_133409 | integrin linked kinase | Ilk | Yes | No | Yes |
| 402 | NM_019127 | interferon beta 1, fibroblast | Ifnb1f | No | N/A | N/A |
| 403 | AF010466 | interferon gamma | Ifng | Yes | No | No |
| 404 | NM_012591 | interferon regulatory factor 1 | Irf1 | Yes | Yes | Yes |
| 405 | NM_017019 | interleukin 1 alpha | Il1a | Yes | No | No |
| 406 | NM_031512 | interleukin 1 beta | Il1b | Yes | Yes | Yes |
| 407 | L02926 | interleukin 10 | Il10 | No | N/A | N/A |
| 408 | AF347936 | interleukin 11 receptor, alpha chain 1 | Il11ra1 | Yes | No | No |
| 409 | NM_053828 | interleukin 13 | Il13 | No | N/A | N/A |
| 410 | AF015718 | interleukin 15 | Il15 | Yes | Yes | Yes |
| 411 | AJ222813 | interleukin 18 | Il18 | Yes | No | Yes |
| 412 | NM_013163 | interleukin 2 receptor, alpha chain | Il2ra | Yes | No | No |
| 413 | NM_013195 | interleukin 2 receptor, beta chain | Il2rb | Yes | No | Yes |
| 414 | NM_031513 | interleukin 3 | Il6 | Yes | No | No |
| 415 | X16058 | interleukin 4 | Il4 | No | N/A | N/A |
| 416 | NM_012589 | interleukin 6 | Il6 | Yes | Yes | Yes |
| 417 | AF367210 | interleukin 7 | Il7 | No | N/A | N/A |
| 418 | BF405951 | Interleukin-1 receptor-associated kinase 4 (predicted) | Irak4_predicted | Yes | Yes | Yes |
| 419 | NM_031514 | Janus kinase 2 | Jak2 | Yes | Yes | Yes |
| 420 | BE096021 | Jun D proto-oncogene | Jund1 | No | N/A | N/A |
| 421 | BI288619 | Jun oncogene | Jun | Yes | Yes | Yes |
| 422 | NM_021836 | Jun-B oncogene | Junb | Yes | Yes | Yes |
| 423 | NM_012696 | kininogen 1 /// K-kininogen /// similar to alpha-1 major acute phase protein prepeptide | LOC25087 | No | N/A | N/A |
| 424 | NM_031135 | Kruppel-like factor 10 | Klf10 | Yes | No | No |
| 425 | BM385790 | Kruppel-like factor 2 (lung) (predicted) | Klf2_predicted | Yes | Yes | Yes |
| 426 | NM_053394 | Kruppel-like factor 5 | Klf5 | Yes | No | No |
| 427 | NM_053902 | kynureninase (L-kynurenine hydrolase) | Kynu | No | N/A | N/A |
| 428 | NM_012594 | Lactalbumin, alpha | Lalba | No | N/A | N/A |
| 429 | NM_019904 | lectin, galactose binding, soluble 1 | Lgals1 | Yes | No | Yes |
| 430 | NM_031832 | lectin, galactose binding, soluble 3 | Lgals3 | Yes | Yes | Yes |
| 431 | NM_022582 | lectin, galactose binding, soluble 7 | Lgals7 | No | N/A | N/A |
| 432 | NM_031048 | leukemia inhibitory factor receptor | Lifr | No | N/A | N/A |
| 433 | NM_031727 | LIM motif-containing protein kinase 1 | Limk1 | No | N/A | N/A |
| 434 | NM_130741 | lipocalin 2 | Lcn2 | Yes | Yes | Yes |
| 435 | BF289368 | lipopolysaccharide binding protein | Lbp | Yes | No | Yes |
| 436 | BI284739 | LPS-induced TN factor | Litaf | Yes | Yes | Yes |
| 437 | AA874924 | lymphocyte antigen 86 (predicted) | Ly86_predicted | Yes | No | Yes |
| 438 | AI137137 | lymphocyte protein tyrosine kinase (mapped) | Lck_mapped | Yes | No | No |
| 439 | AI012109 | lymphocyte specific 1 | Lsp1 | No | N/A | N/A |
| 440 | NM_080769 | lymphotoxin A | Lta | Yes | No | No |
| 441 | NM_053538 | lysosomal-associated protein transmembrane 5 | Laptm5 | Yes | No | No |
| 442 | NM_031051 | macrophage migration inhibitory factor | Mif | Yes | No | Yes |
| **#** | **Accession #** | **NAME** | **Symbol** | **Present** | **Exp. 1** | **Exp. 2** |
| 443 | NM_024352 | Macrophage stimulating 1 (hepatocyte growth factor-like) | Mst1 | No | N/A | N/A |
| 444 | NM_019191 | MAD homolog 2 (Drosophila) | Smad2 | Yes | No | No |
| 445 | AA997679 | MAD homolog 3 (Drosophila) | Smad3 | Yes | No | No |
| 446 | NM_019275 | MAD homolog 4 (Drosophila) | Smad4 | Yes | No | No |
| 447 | AW521447 | MAD homolog 7 (Drosophila) | Madh7 | Yes | Yes | Yes |
| 448 | NM_053585 | MAP-kinase activating death domain | Madd | Yes | No | No |
| 449 | U65656 | matrix metallopeptidase 2 | Mmp2 | Yes | No | No |
| 450 | NM_031055 | matrix metallopeptidase 9 | Mmp9 | No | N/A | N/A |
| 451 | BI289109 | max binding protein (predicted) | Mnt_predicted | Yes | Yes | Yes |
| 452 | AW143154 | megakaryoblastic leukemia (translocation) 1 (predicted) | Mkl1_predicted | Yes | No | No |
| 453 | NM_053409 | melanoma antigen, family D, 1 | Maged1 | Yes | No | No |
| 454 | AF411318 | metallothionein 1a | Mt1a | Yes | Yes | Yes |
| 455 | NM_053307 | methionine sulfoxide reductase A | Msrb2 | Yes | No | No |
| 456 | BI281702 | microtubule-associated protein 1b | Map1b | Yes | No | No |
| 457 | BE107978 | microtubule-associated protein tau /// hypothetical gene supported by NM_017212 | Mapt /// LOC497674 | Yes | No | No |
| 458 | BG665132 | mitochondrial carrier homolog 1 (C. elegans) | Mtch1 | Yes | No | No |
| 459 | AA943734 | mitochondrial protein, 18 kDa | MGC94604 | Yes | Yes | Yes |
| 460 | BG378230 | mitochondrial ribosomal protein S30 (predicted) | Mrps30_predicted | Yes | Yes | Yes |
| 461 | NM_053842 | mitogen activated protein kinase 1 | Mapk1 | Yes | No | No |
| 462 | NM_012806 | mitogen activated protein kinase 10 | Mapk10 | No | N/A | N/A |
| 463 | AW254190 | Mitogen activated protein kinase 14 | Mapk14 | Yes | No | No |
| 464 | AF155236 | mitogen activated protein kinase 3 | Mapk3 | Yes | No | No |
| 465 | NM_053777 | mitogen activated protein kinase 8 interacting protein | Mapk8ip | Yes | No | No |
| 466 | D13341 | mitogen activated protein kinase kinase 1 | Map2k1 | Yes | No | Yes |
| 467 | D14592 | mitogen activated protein kinase kinase 2 | Map2k2 | Yes | No | No |
| 468 | NM_053887 | mitogen activated protein kinase kinase kinase 1 | Map3k1 | Yes | No | No |
| 469 | NM_013055 | mitogen activated protein kinase kinase kinase 12 | Map3k12 | Yes | No | Yes |
| 470 | AI146037 | Mitogen activated protein kinase kinase kinase 7 (predicted) | Map3k7_predicted | Yes | No | No |
| 471 | AI575972 | Mitogen-activated protein kinase 8 interacting protein 2 | Mapk8ip2 | No | N/A | N/A |
| 472 | NM_017322 | mitogen-activated protein kinase 9 | Mapk9 | Yes | No | No |
| 473 | BI281589 | mitogen-activated protein kinase kinase kinase 11 | Map3k11 | Yes | No | No |
| 474 | NM_053847 | mitogen-activated protein kinase kinase kinase 8 | Map3k8 | Yes | Yes | Yes |
| 475 | D00688 | monoamine oxidase A | Maoa | Yes | No | No |
| 476 | NM_012982 | msh homeo box homolog 2 (Drosophila) | Msx2 | No | N/A | N/A |
| 477 | NM_053337 | Msx-interacting-zinc finger | Miz1 | Yes | No | No |
| 478 | BI274326 | mucin 1, transmembrane | Muct1 | No | N/A | N/A |
| 479 | BM391100 | mucin 4 | Muc4 | No | N/A | N/A |
| 480 | NM_031053 | mutL homolog 1 (E. coli) /// hypothetical gene supported by NM_031053 | Mlh1 /// LOC497834 | Yes | No | No |
| 481 | NM_021837 | myc-like oncogene, s-myc protein | Mycs | No | N/A | N/A |
| 482 | NM_012798 | myelin and lymphocyte protein, T-cell differentiation protein | Mal | No | N/A | N/A |
| 483 | BF281184 | myeloblastosis oncogene-like 2 (predicted) | Mybl2_predicted | No | N/A | N/A |
| 484 | NM_012603 | myelocytomatosis viral oncogene homolog (avian) | Myc | Yes | Yes | Yes |
| 485 | AI172056 | myeloid cell leukemia sequence 1 | Mcl1 | Yes | Yes | Yes |
| 486 | BI284349 | myeloid differentiation primary response gene 116 | Myd116 | Yes | Yes | Yes |
| 487 | AI236590 | myeloid differentiation primary response gene 88 | Myd88 | Yes | Yes | Yes |
| 488 | NM_030860 | myocyte enhancer factor 2D | Mef2d | No | N/A | N/A |
| **#** | **Accession #** | **NAME** | **Symbol** | **Present** | **Exp. 1** | **Exp. 2** |
| 489 | J02679 | NAD(P)H dehydrogenase, quinone 1 | Nqo1 | Yes | No | No |
| 490 | NM_053683 | NADPH oxidase 1 | Noxa1 | No | N/A | N/A |
| 491 | AI178285 | NCK-associated protein 1 | Nckap1 | Yes | Yes | No |
| 492 | NM_031069 | NEL-like 1 (chicken) | Nell1 | Yes | No | Yes |
| 493 | NM_012610 | nerve growth factor receptor (TNFR superfamily, member 16) | Ngfr | No | N/A | N/A |
| 494 | NM_053401 | nerve growth factor receptor (TNFRSF16) associated protein 1 | Ngfrap1 | Yes | Yes | No |
| 495 | BM388972 | nerve growth factor, beta (mapped) | Norb | Yes | Yes | No |
| 496 | U02323 | neuregulin 1 | Nrg1 | Yes | No | No |
| 497 | NM_023968 | neuropeptide Y receptor Y2 | Npy2r | No | N/A | N/A |
| 498 | NM_021589 | neurotrophic tyrosine kinase, receptor, type 1 | Ntrk1 | No | N/A | N/A |
| 499 | NM_031073 | neurotrophin 3 | Ntf3 | Yes | No | No |
| 500 | AI598730 | neurotrophin receptor associated death domain | Nradd | Yes | Yes | No |
| 501 | NM_053734 | neutrophil cytosolic factor 1 | Ncf1 | Yes | No | No |
| 502 | BI285459 | nicastrin | Ncstn | Yes | No | Yes |
| 503 | L12562 | nitric oxide synthase 2, inducible | Nos2 | Yes | No | No |
| 504 | AJ011116 | nitric oxide synthase 3, endothelial cell | Nos3 | Yes | No | Yes |
| 505 | NM_053507 | non-metastatic cell expressed protein 3 | Nme3 | Yes | No | No |
| 506 | BF389398 | Notch gene homolog 1 (Drosophila) | Notch1 | Yes | No | No |
| 507 | AI011448 | Notch gene homolog 2 (Drosophila) | Notch2 | Yes | Yes | Yes |
| 508 | NM_020087 | Notch gene homolog 3 (Drosophila) | Notch3 | Yes | No | No |
| 509 | BG377358 | nuclear factor of activated T-cells, cytoplasmic, calcineurin-dependent 4 | Nfatc4 | Yes | No | No |
| 510 | AA858801 | nuclear factor of kappa light chain gene enhancer in B-cells 1, p105 | Nfkb1 | Yes | Yes | No |
| 511 | AW672589 | nuclear factor of kappa light chain gene enhancer in B-cells inhibitor, alpha | Nfkbia | Yes | Yes | Yes |
| 512 | NM_030867 | nuclear factor of kappa light chain gene enhancer in B-cells inhibitor, beta | Nfkbib | Yes | Yes | No |
| 513 | NM_012991 | nuclear pore associated protein | Npap60 | No | N/A | N/A |
| 514 | NM_021745 | nuclear receptor subfamily 1, group H, member 4 | Nr1h4 | No | N/A | N/A |
| 515 | NM_052980 | nuclear receptor subfamily 1, group I, member 2 | Nrc1i2 | Yes | No | No |
| 516 | NM_017323 | nuclear receptor subfamily 2, group C, member 2 | Nrc2c2 | Yes | No | No |
| 517 | AY066016 | nuclear receptor subfamily 3, group C, member 1 | Nr3c1 | Yes | Yes | Yes |
| 518 | NM_013131 | nuclear receptor subfamily 3, group C, member 2 | Nr3c2 | Yes | No | Yes |
| 519 | NM_024388 | nuclear receptor subfamily 4, group A, member 1 | Nr4a1 | Yes | Yes | Yes |
| 520 | NM_031628 | nuclear receptor subfamily 4, group A, member 3 | Nr4a3 | Yes | Yes | Yes |
| 521 | NM_022799 | nuclear ubiquitous casein kinase and cyclin-dependent kinase substrate | Nucks | Yes | No | Yes |
| 522 | NM_053516 | nucleolar protein 3 (apoptosis repressor with CARD domain) | Nol3 | Yes | No | No |
| 523 | NM_012992 | nucleophosmin 1 | Npm1 | Yes | Yes | No |
| 524 | J04943 | nucleophosmin 1 /// similar to Nucleophosmin (NPM) (Nucleolar phosphoprotein B23) (Numatrin) (Nucleolar protein NO38) | Npm1 /// LOC300303 | Yes | No | No |
| 525 | BI286040 | nucleoporin 62 | Nup62 | Yes | No | No |
| 526 | NM_133525 | Nucleoside 2-deoxyribosyltransferase domain containing protein RGD620382 | RGD620382 | No | N/A | N/A |
| 527 | NM_012861 | O-6-methylguanine-DNA methyltransferase | Mgmt | Yes | No | Yes |
| 528 | L20684 | opioid receptor, mu 1 | Oprl1 | Yes | No | No |
| 529 | NM_133585 | optic atrophy 1 homolog (human) | Opa1 | Yes | No | No |
| 530 | NM_053288 | orosomucoid 1 | Orm1 | Yes | Yes | Yes |
| 531 | NM_130402 | osteoclast inhibitory lectin | Ocil | No | N/A | N/A |
| 532 | NM_133306 | oxidized low density lipoprotein (lectin-like) receptor 1 | Oldrlr1 | No | N/A | N/A |
| 533 | NM_019210 | p21 (CDKN1A)-activated kinase 3 | Pak3 | No | N/A | N/A |
| 534 | NM_053289 | pancreatitis-associated protein | Pap | Yes | Yes | Yes |
| **#** | **Accession #** | **NAME** | **Symbol** | **Present** | **Exp. 1** | **Exp. 2** |
| 535 | NM_017044 | parathyroid hormone | Pth | Yes | No | No |
| 536 | BI281756 | Parkinson disease (autosomal recessive, early onset) 7 | Park7 | Yes | Yes | No |
| 537 | BG673589 | paxillin | Pxn | Yes | Yes | No |
| 538 | AI009656 | PEF protein with a long N-terminal hydrophobic domain | Peflin | Yes | No | Yes |
| 539 | BI291292 | peptidylprolyl isomerase C | Ppic | Yes | No | No |
| 540 | AA957342 | peptidylprolyl isomerase D (cyclophilin D) | Ppid | Yes | Yes | No |
| 541 | U68544 | peptidylprolyl isomerase F (cyclophilin F) | Ppif | Yes | Yes | No |
| 542 | NM_017330 | perforin 1 (pore forming protein) | Prf1 | Yes | No | No |
| 543 | NM_017169 | peroxiredoxin 2 | Prdx2 | Yes | No | No |
| 544 | NM_013196 | peroxisome proliferator activated receptor alpha | Ppara | No | N/A | N/A |
| 545 | U75918 | peroxisome proliferator activated receptor delta | Ppard | Yes | No | No |
| 546 | NM_013124 | peroxisome proliferator activated receptor gamma | Pparg | Yes | No | No |
| 547 | AI598971 | PERP, TP53 apoptosis effector (predicted) | Perp_predicted | Yes | Yes | No |
| 548 | NM_021657 | PH domain and leucine rich repeat protein phosphatase | Phlpp | Yes | Yes | Yes |
| 549 | NM_031606 | phosphatase and tensin homolog | Pten | Yes | No | No |
| 550 | NM_053923 | phosphatidylinositol 3-kinase, C2 domain containing, gamma polypeptide | Pik3ca | Yes | No | Yes |
| 551 | BI290699 | phosphatidylinositol 3-kinase, catalytic, alpha polypeptide | Pik3r1 | Yes | No | Yes |
| 552 | D64048 | phosphatidylinositol 3-kinase, regulatory subunit, polypeptide 1 | Pik3r1 | No | N/A | N/A |
| 553 | NM_022185 | phosphatidylinositol 3-kinase, regulatory subunit, polypeptide 2 | Pik3r2 | Yes | No | No |
| 554 | AI103391 | phosphatidylinositol 3-kinase, regulatory subunit, polypeptide 2 | Ptdsrp2 | Yes | No | No |
| 555 | AI232697 | phosphatidylserine receptor | Ptdsr | Yes | No | No |
| 556 | AI454840 | Phosphodiesterase 1A, calmodulin-dependent | Pde1a | Yes | Yes | No |
| 557 | AF327906 | phosphodiesterase 1B, Ca2+calmodulin dependent | Pde1b | No | N/A | N/A |
| 558 | NM_022958 | phosphoinositide-3-kinase, class 3 | Pik3r3 | Yes | Yes | No |
| 559 | NM_133551 | phospholipase A2, group IVA (cytosolic, calcium-dependent) | Pla2g4a | Yes | Yes | Yes |
| 560 | U51898 | phospholipase A2, group VI | Pla2g6 | Yes | No | Yes |
| 561 | U69550 | phospholipase D1 | Pld1 | Yes | No | No |
| 562 | BE112895 | phosphoprotein enriched in astrocytes 15 | Pea15 | Yes | No | Yes |
| 563 | NM_053491 | plasminogen | Plg | No | N/A | N/A |
| 564 | NM_013151 | plasminogen activator, tissue | Plat | Yes | Yes | No |
| 565 | NM_013085 | plasminogen activator, urokinase | Plau | No | N/A | N/A |
| 566 | AF007789 | plasminogen activator, urokinase receptor | Plaur | No | N/A | N/A |
| 567 | BE100812 | Platelet derived growth factor, alpha | Pdgfa | Yes | Yes | No |
| 568 | BM392366 | platelet-activating factor acetylhydrolase, isoform 1b, alpha2 subunit | Pafah1b2 | Yes | Yes | No |
| 569 | BM392366 | platelet-activating factor acetylhydrolase, isoform 1b, alpha2 subunit | Pafah1b2 | Yes | No | No |
| 570 | AI009219 | Pleckstrin homology domain containing, family A member 5 | Plekha5 | Yes | No | No |
| 571 | NM_017180 | pleckstrin homology-like domain, family A, member 1 | Phlda1 | Yes | Yes | Yes |
| 572 | NM_012760 | pleiomorphic adenoma gene-like 1 | Plagl1 | Yes | Yes | No |
| 573 | AB019366 | poly (ADP-ribose) glycohydrolase | Parp1 | Yes | No | No |
| 574 | NM_013063 | poly (ADP-ribose) polymerase family, member 1 | Par1 | Yes | No | No |
| 575 | NM_017141 | polymerase (DNA directed), beta | Polb | Yes | No | Yes |
| 576 | AW531224 | polymerase (RNA) II (DNA directed) polypeptide A (mapped) | Polr2a_mapped | Yes | No | No |
| 577 | AW435212 | potassium channel, subfamily K, member 3 | Kcnk3 | Yes | No | Yes |
| 578 | NM_053405 | potassium channel, subfamily K, member 9 | Kcnk9 | No | N/A | N/A |
| 579 | NM_013186 | potassium voltage gated channel, Shab-related subfamily, member 1 | Kcnb1 | Yes | No | No |
| 580 | BM385544 | presenilin 1 | Psen1 | Yes | No | No |
| **#** | **Accession #** | **NAME** | **Symbol** | **Present** | **Exp. 1** | **Exp. 2** |
| 581 | AB004454 | presenilin 2 | Psen2 | Yes | No | No |
| 582 | AI232272 | presenilin enhancer 2 homolog (C. elegans) | Psenen | Yes | No | Yes |
| 583 | BI278802 | prion protein | Prnp | Yes | No | No |
| 584 | U05989 | PRKC, apoptosis, WT1, regulator | Pawr | Yes | Yes | Yes |
| 585 | BI285575 | procollagen, type 1, alpha 1 | Col1a1 | Yes | No | Yes |
| 586 | BE108058 | Procollagen, type XVIII, alpha 1 | Col18a1 | Yes | No | No |
| 587 | AI599419 | Progesterone receptor | Pgr | Yes | Yes | No |
| 588 | AI704628 | programmed cell death 2 | Pdcd2 | Yes | No | No |
| 589 | NM_022265 | programmed cell death 4 | Pdcd4 | Yes | No | No |
| 590 | BF408447 | programmed cell death 5 (predicted) | Pdcd5_predicted | Yes | No | Yes |
| 591 | BI296393 | programmed cell death 6 (predicted) | Pdcd6_predicted | Yes | No | No |
| 592 | BE328942 | programmed cell death 6 interacting protein | Pdcd6ip | Yes | No | No |
| 593 | AF262320 | programmed cell death 8 | Pdcd8 | Yes | Yes | No |
| 594 | AI013847 | programmed cell death protein 7 (predicted) | Pdcd7_predicted | Yes | No | No |
| 595 | BI282863 | prohibitin | Phb | Yes | No | Yes |
| 596 | NM_012629 | prolactin | Prl | No | N/A | N/A |
| 597 | L48060 | prolactin receptor | Prlr | Yes | No | No |
| 598 | BI290159 | proline-serine-threonine phosphatase-interacting protein 1 (predicted) | Pstpip1_predicted | Yes | No | No |
| 599 | NM_138857 | prominin 2 | Prom2 | Yes | No | No |
| 600 | NM_031644 | prostaglandin D2 synthase 2 | Ptgds2 | Yes | No | No |
| 601 | U03389 | prostaglandin-endoperoxide synthase 2 | Ptgs2 | Yes | Yes | No |
| 602 | AI600136 | protease, serine, 25 | Prss25 | Yes | No | No |
| 603 | NM_031978 | proteasome (prosome, macropain) 26S subunit, non-ATPase, 1 | Psmd1 | Yes | No | No |
| 604 | NM_130430 | proteasome (prosome, macropain) 26S subunit, non-ATPase, 9 | Psmd9 | Yes | No | No |
| 605 | NM_012803 | protein C | Prc | No | N/A | N/A |
| 606 | AI639478 | protein disulfide isomerase associated 2 (predicted) | Pdia2_predicted | Yes | No | Yes |
| 607 | NM_017319 | protein disulfide isomerase associated 3 | Pdia3 | Yes | No | Yes |
| 608 | BF415343 | protein kinase C, alpha | Prkca | Yes | No | No |
| 609 | X04440 | protein kinase C, beta 1 | Prkcb1 | Yes | No | Yes |
| 610 | NM_133307 | protein kinase C, delta | Prkcd | Yes | No | Yes |
| 611 | AA799421 | protein kinase C, epsilon | Prkce | Yes | No | No |
| 612 | NM_019142 | protein kinase, AMP-activated, alpha 1 catalytic subunit | Prkaa1 | Yes | No | Yes |
| 613 | NM_023991 | protein kinase, AMP-activated, alpha 2 catalytic subunit | Prkaa2 | Yes | Yes | No |
| 614 | NM_013012 | protein kinase, cGMP-dependent, type II | Prkg2 | Yes | No | No |
| 615 | BF400782 | protein kinase, DNA activated, catalytic polypeptide (predicted) | Prkdc_predicted | Yes | No | No |
| 616 | NM_019335 | Protein kinase, interferon-inducible double stranded RNA dependent | Prkr | Yes | No | Yes |
| 617 | NM_031527 | protein phosphatase 1, catalytic subunit, alpha isoform | Ppp1ca | Yes | No | No |
| 618 | NM_013065 | protein phosphatase 1, catalytic subunit, beta isoform | Ppp1cb | Yes | No | Yes |
| 619 | NM_022676 | protein phosphatase 1, regulatory (inhibitor) subunit 1A | Ppp1rA | Yes | No | No |
| 620 | AI172276 | protein phosphatase 1, regulatory (inhibitor) subunit 2 | Ppp1r2 | Yes | Yes | Yes |
| 621 | AB023634 | protein phosphatase 1F (PP2C domain containing) | Ppm1f | Yes | No | No |
| 622 | BF408792 | Protein phosphatase 2 (formerly 2A), catalytic subunit, alpha isoform | Ppp2ca | Yes | Yes | No |
| 623 | NM_017040 | protein phosphatase 2 (formerly 2A), catalytic subunit, beta isoform | Ppp2cb | Yes | Yes | No |
| 624 | AA800669 | protein phosphatase 2 (formerly 2A), regulatory subunit A (PR 65), alpha isoform | Ppp2r1a | Yes | No | No |
| 625 | AI717081 | Protein phosphatase 2 (formerly 2A), regulatory subunit B (PR 52), alpha isoform | Ppp2r1b | Yes | Yes | No |
| 626 | BE113127 | Protein phosphatase 3, catalytic subunit, alpha isoform | Ppp3ca | Yes | No | Yes |
| **#** | **Accession #** | **NAME** | **Symbol** | **Present** | **Exp. 1** | **Exp. 2** |
| 627 | NM_031729 | protein phosphatase 5, catalytic subunit | Ppp5c | Yes | No | No |
| 628 | U06230 | protein S (alpha) | Pros1 | Yes | No | No |
| 629 | U69109 | protein tyrosine kinase 2 beta | Ptk2b | No | N/A | N/A |
| 630 | NM_012637 | protein tyrosine phosphatase, non-receptor type 1 | Ptpn1 | Yes | Yes | Yes |
| 631 | AI172465 | Protein tyrosine phosphatase, non-receptor type 11 | Ptpn11 | Yes | No | No |
| 632 | NM_053908 | protein tyrosine phosphatase, non-receptor type 6 | Ptpn6 | Yes | No | No |
| 633 | M10072 | protein tyrosine phosphatase, receptor type, C | Ptprc | Yes | No | No |
| 634 | NM_022925 | protein tyrosine phosphatase, receptor type, Q /// hypothetical gene supported by NM_022925; NM_198323 | Ptprq | Yes | No | No |
| 635 | AI178772 | prothymosin alpha | Ptma | Yes | No | No |
| 636 | NM_017034 | proviral integration site 1 | Pim1 | Yes | No | No |
| 637 | BI294798 | PTK2 protein tyrosine kinase 2 | Ptk2 | Yes | No | No |
| 638 | AF231010 | purinergic receptor P2X, ligand-gated ion channel, 1 | P2rx1 | No | N/A | N/A |
| 639 | AF231010 | purinergic receptor P2X, ligand-gated ion channel, 1 | P2rx1 | No | N/A | N/A |
| 640 | AF020757 | purinergic receptor P2X, ligand-gated ion channel, 2 | P2rx2 | No | N/A | N/A |
| 641 | AF020757 | purinergic receptor P2X, ligand-gated ion channel, 2 | P2rx2 | No | N/A | N/A |
| 642 | NM_019256 | purinergic receptor P2X, ligand-gated ion channel, 7 | P2rx7 | Yes | No | No |
| 643 | NM_017255 | purinergic receptor P2Y, G-protein coupled 2 | P2ry2 | Yes | No | No |
| 644 | BI282953 | PYD and CARD domain containing | Pycard | Yes | No | Yes |
| 645 | NM_013018 | RAB3A, member RAS oncogene family | Rab3a | Yes | No | No |
| 646 | U70777 | rabaptin, RAB GTPase binding effector protein 1 | Rabep1 | Yes | No | No |
| 647 | AJ249986 | Rap guanine nucleotide exchange factor (GEF) 1 | Rapgef1 | No | N/A | N/A |
| 648 | AF002251 | Ras association (RalGDS/AF-6) domain family 5 | Rassf5 | Yes | Yes | No |
| 649 | AF081196 | RAS guanyl releasing protein 1 | Rasgrp2 | Yes | No | No |
| 650 | AI408053 | ras homolog gene family, member A | Rhoa | Yes | No | No |
| 651 | NM_022542 | ras homolog gene family, member B | Rhob | Yes | Yes | Yes |
| 652 | NM_013135 | RAS p21 protein activator 1 | Rasa1 | Yes | Yes | No |
| 653 | BF414025 | Ras-induced senescence 1 | Ris1 | No | N/A | N/A |
| 654 | AA799542 | Ras-related C3 botulinum toxin substrate 1 | Rac1 | Yes | Yes | Yes |
| 655 | AF036537 | receptor-interacting serine-threonine kinase 3 | Ripk3 | Yes | Yes | Yes |
| 656 | NM_012641 | regenerating islet-derived 1 | Reg1 | Yes | No | No |
| 657 | L20869 | regenerating islet-derived 3 gamma | Reg3g | Yes | Yes | Yes |
| 658 | NM_031546 | regucalcin | Rgn | No | N/A | N/A |
| 659 | AJ299017 | ret proto-oncogene | Ret | Yes | No | No |
| 660 | AF051335 | reticulon 4 | Rtn4 | Yes | No | No |
| 661 | AI178012 | retinoblastoma 1 | Rb1 | Yes | Yes | Yes |
| 662 | NM_031094 | retinoblastoma-like 2 | Rbl2 | Yes | No | No |
| 663 | NM_031528 | retinoic acid receptor, alpha | Rara | Yes | No | No |
| 664 | BF419646 | retinoic acid receptor, beta | Rarb | Yes | Yes | No |
| 665 | BI285959 | Retinoid X receptor alpha | Rxra | Yes | Yes | No |
| 666 | AI408677 | Rho GDP dissociation inhibitor (GDI) alpha | Arhgdia | Yes | No | No |
| 667 | NM_031098 | Rho-associated coiled-coil forming kinase 1 | Rock1 | Yes | No | Yes |
| 668 | NM_013022 | Rho-associated coiled-coil forming kinase 2 | Rock1 | No | N/A | N/A |
| 669 | NM_022510 | ribosomal protein L4 | Rpl4 | Yes | Yes | No |
| 670 | BI282255 | ribosomal protein S5 | Rps5 | Yes | No | Yes |
| 671 | M57428 | ribosomal protein S6 kinase, polypeptide 1 | Rps6kb1 | Yes | No | No |
| 672 | AI179991 | ring finger protein 34 | Rnf34 | Yes | No | No |
| **#** | **Accession #** | **NAME** | **Symbol** | **Present** | **Exp. 1** | **Exp. 2** |
| 673 | AA858518 | ring finger protein 7 (predicted) | Rnf7_predicted | Yes | No | Yes |
| 674 | AI175966 | Rous sarcoma oncogene | Src | No | N/A | N/A |
| 675 | BF403180 | Runt related transcription factor 2 | Runx2 | Yes | No | No |
| 676 | NM_012618 | S100 calcium-binding protein A4 | S100a4 | Yes | No | No |
| 677 | NM_013191 | S100 protein, beta polypeptide | S100b | Yes | No | No |
| 678 | AA850867 | sarcoglycan, gamma (dystrophin-associated glycoprotein) | Sgcg | Yes | No | Yes |
| 679 | NM_031541 | scavenger receptor class B, member 1 | Scarb1 | Yes | Yes | No |
| 680 | BI294932 | SCF apoptosis response protein 1 | LOC499941 | Yes | No | No |
| 681 | NM_053687 | schlafen 3 | Slfn3 | Yes | Yes | Yes |
| 682 | BF394953 | SDA1 domain containing 1 | Sdad1 | Yes | No | No |
| 683 | NM_019364 | sec1 family domain containing 1 | Scfd1 | Yes | No | No |
| 684 | AF220608 | secreted frizzled-related protein 4 | Sfrp4 | Yes | No | No |
| 685 | AB001382 | secreted phosphoprotein 1 | Spp1 | Yes | No | Yes |
| 686 | NM_017310 | sema domain, immunoglobulin domain (Ig), short basic domain, secreted, (semaphorin) 3A | Sema3a | No | N/A | N/A |
| 687 | BI299759 | sema domain, transmembrane domain (TM), and cytoplasmic domain, (semaphorin) 6A (predicted) | Sema6a_predicted | No | N/A | N/A |
| 688 | NM_133291 | seminal vesicle antigen-like 2 | Sval2 | No | N/A | N/A |
| 689 | M25590 | seminal vesicle protein 4 | Svp4 | No | N/A | N/A |
| 690 | BG663093 | sequestosome 1 | Sqstm1 | Yes | No | Yes |
| 691 | NM_012620 | serine (or cysteine) peptidase inhibitor, clade E, member 1 | Serpine1 | Yes | Yes | Yes |
| 692 | NM_021696 | serine (or cysteine) proteinase inhibitor, clade B, member 2 | Serpinb2 | No | N/A | N/A |
| 693 | AA944455 | serine incorporator 3 | Serinc3 | Yes | No | Yes |
| 694 | NM_133392 | serine/threonine kinase 17b (apoptosis-inducing) | Stk17b | Yes | No | No |
| 695 | NM_019349 | serine/threonine kinase 2 | Stk2 | Yes | No | Yes |
| 696 | NM_031735 | serine/threonine kinase 3 (STE20 homolog, yeast) | Stk3 | Yes | No | Yes |
| 697 | AF388527 | Serpine1 mRNA binding protein 1 | Serbp1 | Yes | Yes | No |
| 698 | NM_017170 | serum amyloid P-component | Apcs | Yes | No | No |
| 699 | NM_019232 | serum/glucocorticoid regulated kinase | Sgk | Yes | Yes | Yes |
| 700 | NM_080905 | seven in absentia 1A | Siah1a | Yes | No | Yes |
| 701 | NM_134457 | seven in absentia 2 | Siah2 | Yes | No | No |
| 702 | NM_012650 | sex hormone binding globulin | Shbg | Yes | Yes | No |
| 703 | BF284481 | SH3-domain GRB2-like B1 (endophilin) | Sh3glb1 | Yes | No | No |
| 704 | AF255888 | SH3-domain kinase binding protein 1 | Sh3kbp1 | Yes | Yes | Yes |
| 705 | BF550890 | sialophorin | Spn | No | N/A | N/A |
| 706 | NM_032612 | signal transducer and activator of transcription 1 | Stat1 | Yes | No | Yes |
| 707 | BI285863 | signal transducer and activator of transcription 3 | Stat3 | Yes | Yes | Yes |
| 708 | NM_017064 | signal transducer and activator of transcription 5A | Stat5a | Yes | No | No |
| 709 | AI177626 | signal transducer and activator of transcription 5B | Stat5b | Yes | No | No |
| 710 | BE110607 | similar to apoptosis related protein APR-3; p18 protein (predicted) | RGD1311605_predicted | Yes | No | No |
| 711 | BF396386 | similar to cell division cycle and apoptosis regulator 1 (predicted) | RGD1560358_predicted | Yes | Yes | No |
| 712 | BI296385 | similar to chemokine (C-X-C motif) ligand 16 | Cxcl16 | Yes | Yes | Yes |
| 713 | BI294745 | similar to livin inhibitor of apoptosis isoform beta (predicted) | RGD1562883_predicted | Yes | No | No |
| 714 | NM_021846 | similar to MAP/microtubule affinity-regulating kinase 4 (MAP/microtubule affinity-regulating kinase like 1) (predicted) | RGD1561096_predict | Yes | Yes | Yes |
| 715 | AA848545 | similar to programmed cell death 10 | MGC72992 | Yes | No | No |
| 716 | BF282636 | similar to RIKEN cDNA 1700023M03 | RGD1305457 | Yes | No | No |
| **#** | **Accession #** | **NAME** | **Symbol** | **Present** | **Exp. 1** | **Exp. 2** |
| 717 | AW254416 | Similar to TGF-beta induced apoptosis protein 12 (predicted) | RGD1308916_predict | No | N/A | N/A |
| 718 | AA946199 | snail homolog 1 (Drosophila) | Snai1 | Yes | No | No |
| 719 | X89383 | SNF related kinase | Snrk | No | N/A | N/A |
| 720 | NM_012647 | sodium channel, voltage-gated, type 2, alpha 1 polypeptide | Scn2a | No | N/A | N/A |
| 721 | NM_013178 | sodium channel, voltage-gated, type IV, alpha polypeptide | Snc4a | No | N/A | N/A |
| 722 | BI284218 | solute carrier family 2 (facilitated glucose transporter), member 1 | Slc2a1 | Yes | No | Yes |
| 723 | NM_017102 | solute carrier family 2 (facilitated glucose transporter), member 3 | Slc2a3 | Yes | Yes | No |
| 724 | NM_017223 | solute carrier family 20, member 2 | Slc20a2 | Yes | No | Yes |
| 725 | BG666999 | solute carrier family 25 (mitochondrial carrier; adenine nucleotide translocator), member 4 | Slc25a4 | Yes | No | No |
| 726 | NM_017206 | solute carrier family 6 (neurotransmitter transporter, taurine), member 6 | Slc6a6 | Yes | No | No |
| 727 | NM_053442 | solute carrier family 7 (cationic amino acid transporter, y+ system), member 8 /// synaptic Ras GTPase activating protein 1 homolog (rat) | Slc7m8 | No | N/A | N/A |
| 728 | U04933 | solute carrier family 8 (sodium/calcium exchanger), member 1 | Slc7a8 /// Syngap1 | Yes | No | No |
| 729 | NM_133522 | somatostatin receptor 3 /// hypothetical gene supported by NM_133522 | SSTR | Yes | No | No |
| 730 | BI275248 | Son cell proliferation protein | Son | Yes | No | No |
| 731 | NM_017221 | sonic hedgehog homolog (Drosophila) | Shh | No | N/A | N/A |
| 732 | NM_012655 | Sp1 transcription factor | Sp1 | Yes | No | No |
| 733 | AB049572 | sphingosine kinase 1 | Sphk1 | Yes | Yes | No |
| 734 | BM386306 | sphingosine kinase 2 | Sphk2 | Yes | Yes | Yes |
| 735 | AI169638 | sphingosine-1-phosphate phosphatase 1 | Sgpp1 | Yes | No | No |
| 736 | U53883 | ST8 alpha-N-acetyl-neuraminide alpha-2,8-sialyltransferase 1 | St8sia2 | No | N/A | N/A |
| 737 | AY083159 | Stam binding protein | Stambp | Yes | No | No |
| 738 | BM386683 | stanniocalcin 1 | Stc1 | Yes | Yes | Yes |
| 739 | NM_022230 | stanniocalcin 2 | Stc2 | No | N/A | N/A |
| 740 | NM_017166 | stathmin 1 | Stnm1 | Yes | No | No |
| 741 | AF335281 | STEAP family member 3 | Stmn1 | Yes | No | No |
| 742 | AI235465 | steroid sensitive gene 1 | Steap3 | Yes | No | Yes |
| 743 | AI009817 | succinate dehydrogenase complex, subunit C, integral membrane protein | Ssg1 | Yes | No | No |
| 744 | NM_134378 | sulfatase 1 | Sdhc | Yes | No | Yes |
| 745 | NM_017050 | superoxide dismutase 1 | Sod1 | Yes | No | Yes |
| 746 | BG671549 | superoxide dismutase 2, mitochondrial | Sod2 | Yes | Yes | Yes |
| 747 | NM_058208 | suppressor of cytokine signaling 2 | Socs2 | Yes | Yes | Yes |
| 748 | NM_053565 | suppressor of cytokine signaling 3 | Socs3 | Yes | Yes | Yes |
| 749 | BM390864 | survival motor neuron domain containing 1 | Smndc1 | Yes | No | No |
| 750 | AI170385 | SWI/SNF related, matrix associated, actin dependent regulator of chromatin, subfamily a, member 2 | Smarca2 | Yes | No | Yes |
| 751 | BE329013 | SWI/SNF related, matrix associated, actin dependent regulator of chromatin, subfamily a, member 4 | Smarca4 | Yes | Yes | No |
| 752 | NM_053442 | synaptic Ras GTPase activating protein 1 homolog (rat) | Syngap1 | No | N/A | N/A |
| 753 | NM_013026 | syndecan 1 | Sdc1 | Yes | No | No |
| 754 | AA946430 | synovial apoptosis inhibitor 1, synoviolin | Syvn1 | Yes | No | No |
| 755 | NM_019169 | synuclein, alpha | Snca | Yes | No | No |
| 756 | BG671061 | tachykinin 1 | Tac1 | No | N/A | N/A |
| 757 | BM392226 | TAF10 RNA polymerase II, TATA box binding protein (TBP)-associated factor (predicted) | Taf10_predicted | Yes | No | No |
| 758 | NM_133615 | TAF9-like RNA polymerase II, TATA box binding protein (TBP)-associated factor, 31kDa | Taf9l | Yes | No | No |
| 759 | AI228250 | Tax1 (human T-cell leukemia virus type I) binding protein 1 | Tax1bp1 | Yes | No | Yes |
| 760 | AA957545 | T-box 3 | Tbx3 | Yes | Yes | No |
| 761 | AB029495 | TCF3 (E2A) fusion partner | Tfpt | Yes | No | No |
| 762 | AF247818 | telomerase reverse transcriptase | Tert | No | N/A | N/A |
| **#** | **Accession #** | **NAME** | **Symbol** | **Present** | **Exp. 1** | **Exp. 2** |
| 763 | NM_019381 | testis enhanced gene transcript | Tegt | Yes | No | Yes |
| 764 | NM_133396 | testis-specific kinase 2 | Tesk2 | Yes | No | No |
| 765 | AA943723 | THAP domain containing, apoptosis associated protein 3 (predicted) | Thap3_predicted | Yes | No | No |
| 766 | NM_053800 | thioredoxin 1 | Txn1 | Yes | Yes | Yes |
| 767 | AA800180 | thioredoxin 2 | Txn2 | Yes | No | No |
| 768 | BM390196 | thioredoxin domain containing 5 (predicted) | Txndc5_predicted | Yes | No | No |
| 769 | BF555110 | thioredoxin-like 1 | Txnl1 | Yes | Yes | No |
| 770 | BM384228 | THO complex 1 | Thoc1 | Yes | No | No |
| 771 | AA998057 | Thymoma viral proto-oncogene 1 | Akt1 | Yes | No | Yes |
| 772 | AI105076 | thymoma viral proto-oncogene 2 | Akt2 | Yes | No | Yes |
| 773 | NM_031575 | thymoma viral proto-oncogene 3 | Akt3 | No | N/A | N/A |
| 774 | AI145313 | thymus cell antigen 1, theta | Thy1 | Yes | No | No |
| 775 | NM_012888 | thyroid stimulating hormone receptor | Tshr | Yes | Yes | No |
| 776 | AI101391 | Tial1 cytotoxic granule-associated RNA binding protein-like 1 (mapped) | Tial1 | Yes | No | Yes |
| 777 | NM_053819 | tissue inhibitor of metalloproteinase 1 | Timp1 | Yes | Yes | Yes |
| 778 | NM_021989 | tissue inhibitor of metalloproteinase 2 | Timp2 | Yes | No | No |
| 779 | NM_012886 | tissue inhibitor of metalloproteinase 3 (Sorsby fundus dystrophy, pseudoinflammatory) | Timp3 | Yes | No | Yes |
| 780 | AI104533 | titin | Ttn | Yes | No | Yes |
| 781 | BI287742 | TM2 domain containing 1 (predicted) | Tm2d1_predicted | Yes | No | No |
| 782 | BM389034 | TNF receptor-associated protein 1 | Trap1 | Yes | No | No |
| 783 | BM386846 | TNFRSF1A-associated via death domain | Tradd | Yes | No | No |
| 784 | AF057025 | toll-like receptor 4 | Tlr4 | Yes | No | No |
| 785 | AI012419 | Transcribed locus | NS | No | N/A | N/A |
| 786 | AW533194 | Transcribed locus | NS | No | N/A | N/A |
| 787 | AI104523 | Transcribed locus | NS | No | N/A | N/A |
| 788 | AW251860 | transcription factor 7, T-cell specific (predicted) | Tcf7_predicted | Yes | No | Yes |
| 789 | NM_031326 | transcription factor A, mitochondrial | Tfam | Yes | No | No |
| 790 | BI284455 | transcription factor Pur-beta | pur-beta | Yes | No | Yes |
| 791 | NM_133317 | transducer of ErbB-2.1 | Tob1 | Yes | Yes | Yes |
| 792 | M58040 | transferrin receptor | Tfrc | Yes | No | Yes |
| 793 | BI297236 | Transformation related protein 53 inducible nuclear protein 1 | Trp53inp2 | Yes | No | Yes |
| 794 | AJ277449 | transformation related protein 63 | Trp63 | No | N/A | N/A |
| 795 | NM_012671 | transforming growth factor alpha | Tgfa | No | N/A | N/A |
| 796 | AW254561 | transforming growth factor beta regulated gene 4 | Tbrg4 | Yes | No | No |
| 797 | BF420705 | Transforming growth factor, beta 2 | Tgfb2 | Yes | No | No |
| 798 | NM_012775 | transforming growth factor, beta receptor 1 | Tgfbr1 | Yes | No | No |
| 799 | BI275994 | transglutaminase 2, C polypeptide | Tgm2 | Yes | Yes | Yes |
| 800 | AB015231 | transient receptor potential cation channel, subfamily V, member 1 | Trpv1 | No | N/A | N/A |
| 801 | NM_023970 | transient receptor potential cation channel, subfamily V, member 4 /// transient receptor potential cation channel, subfamily V, member 1 | Trpv4 | No | N/A | N/A |
| 802 | AB020967 | tribbles homolog 3 (Drosophila) | Trib3 | Yes | No | Yes |
| 803 | NM_080903 | tripartite motif protein 63 | Trim63 | Yes | Yes | No |
| 804 | AI104913 | tropomodulin 1 | Tmod1 | Yes | No | Yes |
| 805 | NM_031345 | TSC22 domain family 3 | Tsc22d3 | Yes | No | No |
| 806 | BI285434 | tubulin, alpha 1 /// tubulin, alpha 6 /// similar to Tubulin alpha-2 chain (Alpha-tubulin 2) (predicted) | Tuba4a6 | No | N/A | N/A |
| 807 | BI285434 | Tubulin, gamma 1 | Tubg1 | Yes | Yes | Yes |
| 808 | AA819227 | tumor necrosis factor (TNF superfamily, member 2) | Tnf | Yes | No | Yes |
| **#** | **Accession #** | **NAME** | **Symbol** | **Present** | **Exp. 1** | **Exp. 2** |
| 809 | BF283688 | tumor necrosis factor ligand superfamily member 12 | Tnfsf12 | Yes | No | Yes |
| 810 | NM_012870 | tumor necrosis factor receptor superfamily, member 11b (osteoprotegerin) | Tnfrsf11b | No | N/A | N/A |
| 811 | BI303379 | tumor necrosis factor receptor superfamily, member 12a | Tnfrsf12a | Yes | Yes | No |
| 812 | AI169601 | tumor necrosis factor receptor superfamily, member 14 (herpesvirus entry mediator) | Tnfrsf14 | Yes | Yes | No |
| 813 | NM_013091 | tumor necrosis factor receptor superfamily, member 1a | Tnfrsf1a | Yes | Yes | Yes |
| 814 | NM_013049 | tumor necrosis factor receptor superfamily, member 4 | Tnfrsf4 | No | N/A | N/A |
| 815 | AW433947 | tumor necrosis factor receptor superfamily, member 5 | Tnfrsf5 | No | N/A | N/A |
| 816 | NM_139194 | Tumor necrosis factor receptor superfamily, member 6 | Tnfrsf6 | No | N/A | N/A |
| 817 | BM387084 | tumor necrosis factor superfamily, member 5-induced protein 1 (predicted) | Tnfsf5ip1_predicted | Yes | No | Yes |
| 818 | AY009504 | tumor protein p53 | Tp53 | Yes | No | No |
| 819 | NM_053867 | tumor protein, translationally-controlled 1 | Tpt1 | Yes | No | No |
| 820 | BG057543 | tumor rejection antigen gp96 (predicted) | Tra1_predicted | Yes | Yes | Yes |
| 821 | AI234654 | tumor susceptibility gene 101 | Tsg101 | Yes | No | No |
| 822 | NM_013052 | tyrosine 3-monooxygenase/tryptophan 5-monooxygenase activation protein, eta polypeptide | Ywhah | Yes | No | No |
| 823 | NM_019376 | tyrosine 3-monooxygenase/tryptophan 5-monooxygenase activation protein, gamma polypeptide | Ywhag | Yes | No | Yes |
| 824 | BF281342 | tyrosine 3-monooxygenase/tryptophan 5-monooxygenase activation protein, theta polypeptide | Ywhaq | Yes | Yes | No |
| 825 | AI228292 | tyrosine hydroxylase | Th | Yes | No | No |
| 826 | AI407490 | tyrosyl-tRNA synthetase | Yars | Yes | Yes | Yes |
| 827 | NM_053747 | ubiquilin 1 | Ubqln1 | Yes | Yes | Yes |
| 828 | BI276086 | Ubiquitin specific protease 7 (herpes virus-associated) | Usp7 | Yes | No | No |
| 829 | BI296848 | ubiquitination factor E4B, UFD2 homolog (S. cerevisiae) (predicted) | Ube4b_predicted | Yes | No | No |
| 830 | AI102437 | ubiquitin-like 1 (sentrin) activating enzyme E1B | Uble1b | Yes | No | No |
| 831 | AF159706 | unc-13 homolog B (C. elegans) | Unc13b | Yes | No | No |
| 832 | NM_022206 | unc-5 homolog A (C. elegans) | Unc5a | No | N/A | N/A |
| 833 | NM_022207 | unc-5 homolog B (C. elegans) | Unc5b | Yes | No | No |
| 834 | BM388453 | uncharacterized protein family UPF0227 member RGD1359682 | RGD1359682 | Yes | No | No |
| 835 | BM388453 | uncharacterized protein family UPF0227 member RGD1359682 | RGD1359682 | No | N/A | N/A |
| 836 | U30789 | upregulated by 1,25-dihydroxyvitamin D-3 | Txnip | Yes | Yes | No |
| 837 | U30789 | upregulated by 1,25-dihydroxyvitamin D-3 | Uvdr | No | N/A | N/A |
| 838 | NM_053864 | valosin-containing protein | Vcp | Yes | No | No |
| 839 | AF080594 | vascular endothelial growth factor A | Vegfa | Yes | No | No |
| 840 | NM_012759 | vav 1 oncogene | Vav1 | Yes | No | No |
| 841 | NM_021687 | v-erb-a erythroblastic leukemia viral oncogene homolog 4 (avian) | Erbb4 | No | N/A | N/A |
| 842 | NM_017003 | v-erb-b2 erythroblastic leukemia viral oncogene homolog 2, neuro/glioblastoma derived oncogene homolog (avian) | Erbb2 | Yes | No | Yes |
| 843 | NM_012555 | v-ets erythroblastosis virus E26 oncogene homolog 1 (avian) | Ets1 | Yes | Yes | Yes |
| 844 | NM_031140 | vimentin | Vim | Yes | Yes | No |
| 845 | AF268467 | voltage-dependent anion channel 1 | Vdac1 | Yes | Yes | Yes |
| 846 | NM_012639 | v-raf-1 murine leukemia viral oncogene homolog 1 | Raf1 | Yes | No | No |
| 847 | BF283772 | v-rel reticuloendotheliosis viral oncogene homolog A (avian) | Rela | Yes | Yes | No |
| 848 | NM_022548 | wild-type p53-induced gene 1 | Wig1 | Yes | No | No |
| 849 | NM_031534 | Wilms tumor 1 | Wt1 | Yes | No | No |
| 850 | NM_031590 | WNT1 inducible signaling pathway protein 2 | Wisp2 | Yes | No | No |
| 851 | BI300732 | WW domain-containing oxidoreductase (predicted) | RGD1565791_predicted | Yes | No | No |
| 852 | BI282111 | Y box protein 1 related, pseudogene 3 /// similar to nuclease sensitive element binding protein 1 (predicted) /// Y box protein 1 | Ybx1-ps3 / RGD1560265_predict/ Ybx1 | Yes | No | No |
| **#** | **Accession #** | **NAME** | **Symbol** | **Present** | **Exp. 1** | **Exp. 2** |
| 853 | NM_031615 | zinc finger protein 148 | Zfp148 | Yes | No | No |
| 854 | BE109605 | zinc finger protein 162 | Zfp162 | Yes | Yes | No |
| 855 | BE111799 | Zinc finger protein 346 (predicted) | Zfp346_predicted | No | N/A | N/A |
| 856 | BM392399 | zinc finger protein 622 | Zfp622 | Yes | No | No |
| 857 | AA819804 | Zinc finger protein 91 | Zfp91 | Yes | No | No |
| 858 | BI289543 | zinc finger, MYND domain containing 11 | Zmynd11 | Yes | Yes | No |
| 859 | AI317860 | zinc responsive protein ZD7 | LOC474154 | Yes | No | No |

* Signal detected above background for gene probeset in 20% or more of the chips.

^†^ “Yes” indicates significant differential gene expression within the Sham, IHS/P, and IHS/I groups (Exp. 1); or within the IHS/I/NS-ODN and IHS/I/GQ-ODN groups (Exp. 2) using False Discovery Rate (FDR) = 10%. “No” indicates not significant differential gene expression. “N/A” indicates genes not included in the analysis because of not being detected in at least 20% of the chips. “NS” indicates no gene symbol.
