# Supplementary material for: Prevention of Hypovolemic Circulatory Collapse by IL-6 Activated Stat3
Source: PLoS One. 2008 Feb 13;3(2):e1605. doi: 10.1371/journal.pone.0001605 (PMC2225503; doi:10.1371/journal.pone.0001605)
Supplement: Table S2 — Supplemental Table 2 (0.38 MB DOC) [file pone.0001605.s002.doc]

**Table S2.** Apoptosis pathway genes whose expression is altered in severe hemorrhagic shock.

| **mRNA**  **Accesion#** | **Gene Name^A^** | **Gene**  **Symbol** | **SBR50/P vs. Sham** | | **SBR50/IL-6 vs.**  **SBR50** | | **SBR50/IL-6/GQ vs.**  **SBR50/IL-6/NS** | |
| --- | --- | --- | --- | --- | --- | --- | --- | --- |
|  |  |  | **Fold**  **P/Sham** | **FDR^B^** | **Fold**  **IL-6/P** | **FDR^B^** | **Fold**  **GQ/NS** | **FDR^B^** |
| **GROUP IA GENES INCREASED IN PBS VS SHAM AND DECREASED IN IL6 VS PBS COMPARISONS** | | | | | | |  |  |
| AA818262 | angiopoietin-like 4 | Angptl4 | 75.19 | 0.0000 | 0.37 | 0.0893 | 31.91 | 0.0000 |
| NM_012912 | activating transcription factor 3 | Atf3 | 33.07 | 0.0000 | 0.17 | 0.0015 | 4.42 | 0.0005 |
| BI278231 | heat shock 70kD protein 1B (mapped) | Hspa1b | 22.12 | 0.0000 | 0.09 | 0.0002 | 1.10 | 0.8340 |
| NM_031971 | heat shock 70kD protein 1A /// heat shock 70kD protein 1B (mapped) | Hspa1a | 20.50 | 0.0000 | 0.10 | 0.0018 | 1.63 | 0.3438 |
| BM384926 | DnaJ (Hsp40) homolog, subfamily B, member 1 (predicted) | Dnajb1 | 8.63 | 0.0000 | 0.20 | 0.0028 | 2.02 | 0.0766 |
| AB003726 | homer homolog 1 (Drosophila) | Homer1 | 8.10 | 0.0007 | 0.16 | 0.0123 | 0.95 | 0.9374 |
| NM_024125 | CCAAT/enhancer binding protein (C/EBP), beta | Cebpb | 6.86 | 0.0000 | 0.53 | 0.0123 | 3.46 | 0.0000 |
| AI236590 | myeloid differentiation primary response gene 88 | Myd88 | 5.86 | 0.0000 | 0.61 | 0.0760 | 2.83 | 0.0001 |
| NM_031514 | Janus kinase 2 | Jak2 | 3.42 | 0.0002 | 0.40 | 0.0123 | 1.98 | 0.0177 |
| AI236601 | heat shock 105kDa/110kDa protein 1 | Hsph1 | 3.36 | 0.0006 | 0.34 | 0.0108 | 0.85 | 0.6286 |
| AI172056 | myeloid cell leukemia sequence 1 | Mcl1 | 3.14 | 0.0000 | 0.64 | 0.0383 | 1.74 | 0.0037 |
| BI284349 | myeloid differentiation primary response gene 116 | Myd116 | 3.00 | 0.0000 | 0.60 | 0.0167 | 2.01 | 0.0004 |
| BM389208 | GTPase, IMAP family member 4 | Gimap4 | 2.63 | 0.0033 | 0.39 | 0.0186 | 1.70 | 0.0907 |
| NM_012954 | fos-like antigen 2 /// FBJ osteosarcoma oncogene B | Fosl2 | 2.61 | 0.0003 | 0.43 | 0.0085 | 1.78 | 0.0161 |
| NM_031327 | cysteine rich protein 61 | Cyr61 | 2.54 | 0.0238 | 0.38 | 0.0565 | 1.43 | 0.3998 |
| BG671521 | heat shock protein 1, alpha | Hspca | 2.32 | 0.0001 | 0.55 | 0.0123 | 0.89 | 0.5640 |
| NM_053747 | ubiquilin 1 | Frap1 | 2.12 | 0.0001 | 0.67 | 0.0335 | 1.38 | 0.0360 |
| BI296385 | similar to chemokine (C-X-C motif) ligand 16 | Cxcl16 | 2.01 | 0.0008 | 0.58 | 0.0241 | 2.49 | 0.0001 |
| NM_012715 | adrenomedullin | Adm | 1.49 | 0.0632 | 0.55 | 0.0289 | 3.11 | 0.0001 |
| BM386683 | stanniocalcin 1 | Stc1 | 1.40 | 0.0056 | 0.77 | 0.0838 | 1.25 | 0.0604 |
| BG057543 | tumor rejection antigen gp96 (predicted) | Tra1 | 1.38 | 0.0024 | 0.74 | 0.0167 | 1.33 | 0.0069 |
| *NM_031628* | *nuclear receptor subfamily 4, group A, member 3* | *Nr4a3* | *48.25* | *0.0000* | *0.22* | *0.0252* | *2.67* | *0.0650* |
| *NM_024388* | *nuclear receptor subfamily 4, group A, member 1* | *Nr4a1* | *19.09* | *0.0000* | *0.49* | *0.0494* | *3.49* | *0.0004* |
| *AI176519* | *immediate early response 3* | *Ier3* | *17.36* | *0.0000* | *0.31* | *0.0491* | *5.94* | *0.0010* |
| *BI284739* | *LPS-induced TN factor* | *Litaf* | *7.62* | *0.0000* | *0.48* | *0.0018* | *3.23* | *0.0000* |
| *NM_133578* | *dual specificity phosphatase 5* | *Dusp5* | *6.19* | *0.0000* | *0.32* | *0.0108* | *2.09* | *0.0321* |
| *AA858801* | *nuclear factor of kappa light chain gene enhancer in B-cells 1, p105* | *Nfkb1* | *4.15* | *0.0000* | *0.47* | *0.0325* | *1.35* | *0.2952* |
| *NM_012950* | *coagulation factor II (thrombin) receptor* | *F2r* | *3.65* | *0.0000* | *0.64* | *0.0491* | *1.53* | *0.0225* |
| *NM_053319* | *dynein light chain LC8-type 1* | *Dynll1* | *2.98* | *0.0001* | *0.52* | *0.0279* | *0.82* | *0.4289* |
| *NM_024356* | *GTP cyclohydrolase 1* | *Gch* | *2.75* | *0.0012* | *0.40* | *0.0149* | *3.39* | *0.0004* |
| *NM_053847* | *mitogen-activated protein kinase kinase kinase 8* | *Map3k8* | *2.71* | *0.0002* | *0.51* | *0.0241* | *2.96* | *0.0002* |
| *NM_021846* | *similar to MAP/microtubule affinity-regulating kinase 4 (MAP/microtubule affinity-regulating kinase like 1) (predicted)* | *RGD1561096* | *2.62* | *0.0000* | *0.64* | *0.0235* | *2.64* | *0.0000* |
| *NM_012760* | *pleiomorphic adenoma gene-like 1* | *Plagl1* | *1.93* | *0.0001* | *0.61* | *0.0108* | *0.84* | *0.2079* |
| *NM_012637* | *protein tyrosine phosphatase, non-receptor type 1* | *Ptpns1* | *1.90* | *0.0000* | *0.79* | *0.0904* | *1.48* | *0.0013* |
| *AF002251* | *Ras association (RalGDS/AF-6) domain family 5* | *Rassf5* | *1.39* | *0.0092* | *0.67* | *0.0123* | *1.13* | *0.3201* |
|  |  |  |  |  |  |  |  |  |
| **GROUP IB GENES INCREASED IN PBS VS SHAM AND UNCHANGED IN IL6 VS PBS COMPARISON** | | | | | | |  |  |
| NM_012589 | interleukin 6 | Il6 | 26.17 | 0.0011 | 0.26 | 0.3038 | 5.13 | 0.0759 |
| NM_031530 | chemokine (C-C motif) ligand 2 | Ccl2 | 25.28 | 0.0010 | 0.25 | 0.2781 | 18.68 | 0.0030 |
| NM_012603 | myelocytomatosis viral oncogene homolog (avian) | Myc | 22.78 | 0.0000 | 0.66 | 0.3199 | 13.00 | 0.0000 |
| BI288701 | B-cell translocation gene 2, anti-proliferative | Btg2 | 13.27 | 0.0000 | 0.70 | 0.2141 | 9.15 | 0.0000 |
| NM_021836 | Jun-B oncogene | Junb | 12.71 | 0.0000 | 0.82 | 0.5914 | 13.18 | 0.0000 |
| NM_013154 | CCAAT/enhancer binding protein (C/EBP), delta | Cebpd | 11.81 | 0.0000 | 0.53 | 0.1437 | 15.45 | 0.0000 |
| NM_053565 | suppressor of cytokine signaling 3 | Socs3 | 8.50 | 0.0000 | 0.84 | 0.8880 | 20.48 | 0.0000 |
| NM_012620 | serine (or cysteine) peptidase inhibitor, clade E, member 1 | Serpine1 | 8.40 | 0.0000 | 0.89 | 0.8880 | 13.57 | 0.0000 |
| AF411318 | metallothionein 1a | Mt1a | 7.25 | 0.0000 | 1.05 | 0.9686 | 18.98 | 0.0000 |
| NM_017334 | cAMP responsive element modulator | Crem | 6.89 | 0.0000 | 0.65 | 0.2781 | 1.84 | 0.0321 |
| L20869 | regenerating islet-derived 3 gamma | Reg3g | 6.17 | 0.0377 | 0.50 | 0.7073 | 10.35 | 0.0118 |
| NM_023987 | baculoviral IAP repeat-containing 3 | Birc3 | 3.75 | 0.0092 | 0.38 | 0.1332 | 3.79 | 0.0106 |
| BI285863 | signal transducer and activator of transcription 3 | Stat3 | 2.95 | 0.0000 | 0.79 | 0.2398 | 3.46 | 0.0000 |
| NM_021752 | baculoviral IAP repeat-containing 2 | Birc2 | 2.80 | 0.0005 | 0.59 | 0.1332 | 1.66 | 0.0585 |
| NM_031970 | heat shock 27kDa protein 1 | Hspb1 | 2.75 | 0.0005 | 0.70 | 0.3589 | 2.34 | 0.0030 |
| NM_058208 | suppressor of cytokine signaling 2 | Socs2 | 2.69 | 0.0000 | 0.70 | 0.1332 | 1.66 | 0.0089 |
| NM_031328 | B-cell CLL/lymphoma 10 | Bcl10 | 2.66 | 0.0004 | 0.79 | 0.6029 | 2.74 | 0.0004 |
| BI288619 | Jun oncogene | Jun | 2.63 | 0.0004 | 0.96 | 0.9689 | 3.29 | 0.0001 |
| NM_053288 | orosomucoid 1 | Orm1 | 2.57 | 0.0172 | 1.08 | 0.9686 | 14.33 | 0.0000 |
| NM_019232 | serum/glucocorticoid regulated kinase | Sgk | 2.33 | 0.0006 | 1.46 | 0.2123 | 2.32 | 0.0010 |
| NM_013151 | plasminogen activator, tissue | Plat | 2.13 | 0.0012 | 0.84 | 0.7073 | 0.90 | 0.6639 |
| BI294137 | Hexokinase 2 | Hk2 | 2.13 | 0.0012 | 0.68 | 0.1838 | 1.41 | 0.1074 |
| NM_053843 | Fc receptor, IgG, low affinity III /// Fc gamma receptor II beta | Fcgr3 | 2.10 | 0.0279 | 0.71 | 0.5914 | 2.86 | 0.0041 |
| BF408792 | Protein phosphatase 2 (formerly 2A), catalytic subunit, alpha isoform | Ppp2ca | 1.99 | 0.0004 | 1.15 | 0.7073 | 1.03 | 0.8783 |
| NM_022542 | ras homolog gene family, member B | Rhob | 1.98 | 0.0000 | 0.83 | 0.3199 | 1.83 | 0.0003 |
| AF228684 | adenosine A2a receptor | Adora2a | 1.86 | 0.0184 | 0.71 | 0.3780 | 1.31 | 0.3075 |
| BF419646 | retinoic acid receptor, beta | Rarb | 1.85 | 0.0129 | 0.70 | 0.3049 | 0.83 | 0.4677 |
| NM_017022 | integrin beta 1 (fibronectin receptor beta) | Itgb1 | 1.83 | 0.0000 | 0.96 | 0.9059 | 1.59 | 0.0004 |
| NM_024359 | hypoxia inducible factor 1, alpha subunit | Hif1a | 1.76 | 0.0362 | 0.70 | 0.3780 | 1.07 | 0.8343 |
| M12672 | guanine nucleotide binding protein, alpha inhibiting 2 | Gnai2 | 1.71 | 0.0075 | 0.82 | 0.5914 | 1.00 | 0.9903 |
| BE100812 | Platelet derived growth factor, alpha | Pdgfa | 1.68 | 0.0762 | 0.58 | 0.1619 | 0.87 | 0.6823 |
| BM388972 | nerve growth factor, beta (mapped) | Ngfb | 1.68 | 0.0119 | 0.69 | 0.1619 | 1.02 | 0.9311 |
| AI230294 | Peroxisome proliferator activated receptor delta | Ppard | 1.66 | 0.0208 | 0.99 | 0.9924 | 1.34 | 0.1817 |
| NM_012931 | breast cancer anti-estrogen resistance 1 | Bcar1 | 1.64 | 0.0767 | 1.34 | 0.5914 | 1.73 | 0.0525 |
| BI285434 | tubulin, alpha 1 /// tubulin, alpha 6 /// similar to Tubulin alpha-2 chain (Alpha-tubulin 2) (predicted) | Tuba1 | 1.62 | 0.0012 | 0.82 | 0.3197 | 0.69 | 0.0116 |
| AW672589 | nuclear factor of kappa light chain gene enhancer in B-cells inhibitor, alpha | Nfkbia | 1.57 | 0.0056 | 0.80 | 0.3167 | 2.80 | 0.0000 |
| NM_022399 | calreticulin | Calr | 1.57 | 0.0061 | 0.73 | 0.1332 | 1.60 | 0.0060 |
| BE109605 | zinc finger protein 162 | Zfp162 | 1.55 | 0.0289 | 0.92 | 0.9042 | 1.09 | 0.7028 |
| BM392366 | platelet-activating factor acetylhydrolase, isoform 1b, alpha2 subunit | Pafah1b2 | 1.54 | 0.0205 | 0.79 | 0.4035 | 0.94 | 0.7722 |
| AW433973 | craniofacial development protein 1 | Cfdp1 | 1.53 | 0.0230 | 1.14 | 0.7491 | 1.07 | 0.7454 |
| AI172276 | protein phosphatase 1, regulatory (inhibitor) subunit 2 | Ppp1r2 | 1.53 | 0.0248 | 0.90 | 0.8679 | 0.93 | 0.7374 |
| AI012221 | chloride intracellular channel 1 | Clic1 | 1.51 | 0.0075 | 0.89 | 0.7165 | 2.03 | 0.0002 |
| U68544 | peptidylprolyl isomerase F (cyclophilin F) | Ppif | 1.50 | 0.0329 | 0.92 | 0.8885 | 1.12 | 0.5779 |
| BI296087 | Serpine1 mRNA binding protein 1 | Serbp1 | 1.46 | 0.0411 | 0.83 | 0.5914 | 0.81 | 0.2568 |
| AA957342 | peptidylprolyl isomerase D (cyclophilin D) | Ppid | 1.44 | 0.0006 | 0.86 | 0.2819 | 1.10 | 0.3348 |
| BF396386 | similar to cell division cycle and apoptosis regulator 1 (predicted) | RGD1560358 | 1.43 | 0.0458 | 0.83 | 0.5822 | 1.02 | 0.9335 |
| BM383722 | NCK-associated protein 1 | Nckap1 | 1.43 | 0.0377 | 0.81 | 0.4373 | 0.97 | 0.8783 |
| AB015946 | tubulin, gamma 1 | Tubg1 | 1.40 | 0.0632 | 0.88 | 0.7693 | 0.80 | 0.2344 |
| NM_031832 | lectin, galactose binding, soluble 3 | Lgals3 | 1.40 | 0.0068 | 1.22 | 0.2269 | 2.04 | 0.0000 |
| AI103616 | ras-related C3 botulinum toxin substrate 1 | Rac1 | 1.39 | 0.0498 | 0.87 | 0.7073 | 1.61 | 0.0089 |
| AI178012 | retinoblastoma 1 | Rb1 | 1.38 | 0.0719 | 0.90 | 0.8274 | 0.65 | 0.0239 |
| BF281342 | tyrosine 3-monooxygenase/tryptophan 5-monooxygenase activation protein, theta polypeptide | Ywhaq | 1.38 | 0.0471 | 0.92 | 0.8807 | 0.78 | 0.1413 |
| M14050 | heat shock 70kDa protein 5 (glucose-regulated protein) | Hspa5 | 1.37 | 0.0273 | 0.82 | 0.3167 | 1.44 | 0.0147 |
| AI237389 | heat shock 90kDa protein 1, beta | Hspcb | 1.34 | 0.0116 | 0.81 | 0.1619 | 1.03 | 0.7874 |
| NM_030867 | nuclear factor of kappa light chain gene enhancer in B-cells inhibitor, beta | Nfkbib | 1.33 | 0.0971 | 0.86 | 0.6769 | 1.03 | 0.8817 |
| NM_017102 | solute carrier family 2 (facilitated glucose transporter), member 3 | Slc2a3 | 1.30 | 0.0111 | 0.82 | 0.1245 | 0.89 | 0.2331 |
| NM_031140 | vimentin | Vim | 1.17 | 0.0767 | 1.01 | 0.9734 | 0.94 | 0.5191 |
| BE108192 | G1 to S phase transition 1 | Gspt1 | 1.15 | 0.0934 | 0.88 | 0.3167 | 0.81 | 0.0182 |
| *NM_012551* | *early growth response 1* | *Egr1* | *52.07* | *0.0000* | *0.96* | *0.9734* | *14.33* | *0.0000* |
| *NM_130741* | *lipocalin 2* | *Lcn2* | *24.85* | *0.0000* | *1.11* | *0.9633* | *21.42* | *0.0000* |
| *AI599423* | *growth arrest and DNA-damage-inducible 45 gamma* | *Gadd45g* | *14.25* | *0.0000* | *1.05* | *0.9720* | *15.60* | *0.0000* |
| *NM_053819* | *tissue inhibitor of metalloproteinase 1* | *Timp1* | *13.41* | *0.0000* | *0.66* | *0.5914* | *5.91* | *0.0003* |
| *NM_021744* | *CD14 antigen* | *Cd14* | *13.36* | *0.0000* | *0.43* | *0.1188* | *9.91* | *0.0000* |
| *NM_012580* | *heme oxygenase (decycling) 1* | *Hmox1* | *7.36* | *0.0018* | *0.55* | *0.5914* | *5.75* | *0.0066* |
| *NM_031512* | *interleukin 1 beta* | *Il1b* | *7.03* | *0.0105* | *0.35* | *0.3167* | *15.83* | *0.0011* |
| *NM_053289* | *pancreatitis-associated protein* | *Pap* | *4.03* | *0.0490* | *0.67* | *0.8679* | *9.69* | *0.0037* |
| *BI303379* | *tumor necrosis factor receptor superfamily, member 12a* | *Tnfrsf12a* | *3.76* | *0.0002* | *0.96* | *0.9734* | *1.21* | *0.5756* |
| *AB049572* | *sphingosine kinase 1* | *Sphk1* | *3.69* | *0.0095* | *0.38* | *0.1332* | *1.25* | *0.6843* |
| *NM_017180* | *pleckstrin homology-like domain, family A, member 1* | *Phlda1* | *3.33* | *0.0011* | *0.58* | *0.2398* | *2.04* | *0.0381* |
| *NM_012591* | *interferon regulatory factor 1* | *Irf1* | *2.85* | *0.0184* | *0.58* | *0.4184* | *4.45* | *0.0022* |
| *NM_017258* | *B-cell translocation gene 1, anti-proliferative* | *Btg1* | *2.85* | *0.0000* | *0.67* | *0.1245* | *1.55* | *0.0319* |
| *NM_013091* | *tumor necrosis factor receptor superfamily, member 1a* | *Tnfrsf1a* | *2.52* | *0.0001* | *0.93* | *0.9103* | *3.78* | *0.0000* |
| *BF283772* | *v-rel reticuloendotheliosis viral oncogene homolog A (avian)* | *Rela* | *2.07* | *0.0245* | *0.77* | *0.7028* | *1.47* | *0.2346* |
| *U03389* | *prostaglandin-endoperoxide synthase 2* | *Ptgs2* | *2.05* | *0.0383* | *0.77* | *0.7295* | *1.40* | *0.3459* |
| *U05989* | *PRKC, apoptosis, WT1, regulator* | *Pawr* | *1.99* | *0.0289* | *0.74* | *0.6119* | *2.31* | *0.0113* |
| *AI103600* | *Filamin C, gamma (actin binding protein 280) (predicted)* | *Flnc* | *1.99* | *0.0042* | *0.92* | *0.9059* | *1.17* | *0.5163* |
| *AI010427* | *cyclin-dependent kinase inhibitor 1A* | *Cdkn1a* | *1.91* | *0.0000* | *1.07* | *0.7491* | *1.68* | *0.0001* |
| *X57764* | *endothelin receptor type B* | *Ednrb* | *1.84* | *0.0317* | *0.68* | *0.3650* | *1.82* | *0.0381* |
| *L07268* | *aquaporin 1* | *Aqp1* | *1.72* | *0.0012* | *0.87* | *0.6321* | *1.34* | *0.0599* |
| *AI231792* | *Bcl2-associated athanogene 3* | *Bag3* | *1.69* | *0.0038* | *0.82* | *0.4866* | *1.02* | *0.9256* |
| *AF000942* | *inhibitor of DNA binding 3* | *Id3* | *1.63* | *0.0075* | *1.09* | *0.8824* | *1.43* | *0.0465* |
| *NM_024134* | *DNA-damage inducible transcript 3* | *Ddit3* | *1.61* | *0.0012* | *0.76* | *0.1332* | *2.29* | *0.0000* |
| *AI598971* | *PERP, TP53 apoptosis effector (predicted)* | *Perp* | *1.60* | *0.0540* | *0.85* | *0.7917* | *1.40* | *0.1734* |
| *BF403027* | *histone deacetylase 5* | *Hdac5* | *1.49* | *0.0248* | *0.73* | *0.1781* | *0.83* | *0.2983* |
| *BG671549* | *superoxide dismutase 2, mitochondrial* | *Sod2* | *1.48* | *0.0172* | *0.79* | *0.3049* | *1.39* | *0.0444* |
| *NM_053883* | *dual specificity phosphatase 6* | *Dusp6* | *1.42* | *0.0239* | *1.03* | *0.9720* | *2.21* | *0.0001* |
| *NM_017040* | *protein phosphatase 2 (formerly 2A), catalytic subunit, beta isoform* | *Ppp2cb* | *1.36* | *0.0195* | *0.90* | *0.7073* | *0.82* | *0.1202* |
| *AF036537* | *receptor-interacting serine-threonine kinase 3* | *Ripk3* | *1.32* | *0.0709* | *0.73* | *0.1245* | *2.16* | *0.0001* |
| *BF555110* | *thioredoxin-like 1* | *Txnl1* | *1.32* | *0.0541* | *0.98* | *0.9734* | *0.96* | *0.7982* |
| *BI275994* | *transglutaminase 2, C polypeptide* | *Tgm2* | *1.31* | *0.0409* | *0.88* | *0.6269* | *1.48* | *0.0069* |
| *NM_031541* | *scavenger receptor class B, member 1* | *Scarb1* | *1.31* | *0.0966* | *0.74* | *0.1619* | *1.14* | *0.4562* |
| *BI280304* | *Bcl2-associated athanogene 1 (predicted)* | *Bag1* | *1.30* | *0.0593* | *1.00* | *0.9924* | *1.09* | *0.5589* |
| *AI407490* | *tyrosyl-tRNA synthetase* | *Yars* | *1.28* | *0.0767* | *0.86* | *0.5451* | *2.18* | *0.0000* |
| *BF281311* | *casein kinase 2, beta subunit* | *Csnk2b* | *1.20* | *0.0559* | *0.96* | *0.8967* | *0.99* | *0.9304* |
| *NM_012992* | *nucleophosmin 1* | *Npm1* | *1.20* | *0.0343* | *0.94* | *0.7304* | *1.10* | *0.2835* |
| *NM_053800* | *thioredoxin 1* | *Txn1* | *1.19* | *0.0364* | *1.00* | *0.9924* | *1.56* | *0.0000* |
| *NM_022510* | *ribosomal protein L4* | *Rpl4* | *1.15* | *0.0134* | *0.99* | *0.9621* | *1.00* | *0.9547* |
| *L81174* | *ankyrin repeat domain 1 (cardiac muscle)* | *Ankrd1* | *1.12* | *0.0762* | *0.91* | *0.2986* | *1.03* | *0.6839* |
| *NM_012839* | *cytochrome c, somatic* | *Cycs* | *1.09* | *0.0708* | *1.00* | *0.9832* | *0.91* | *0.0509* |
|  |  |  |  |  |  |  |  |  |
| **GROUP IIA GENES DECREASED IN PBS VS SHAM AND INCREASED IN IL6 VS PBS COMPARISON** | | | | | | |  |  |
| NM_022407 | aldehyde dehydrogenase family 1, member A1 | Aldh1a1 | 0.62 | 0.0611 | 2.06 | 0.0279 | 0.65 | 0.1047 |
| BI285682 | DnaJ (Hsp40) homolog, subfamily C, member 7 | Dnajc7 | 0.72 | 0.0027 | 1.32 | 0.0335 | 1.04 | 0.7547 |
| *NM_133317* | *transducer of ErbB-2.1* | *Tob1* | *0.16* | *0.0000* | *3.07* | *0.0028* | *0.63* | *0.0907* |
| *NM_080903* | *tripartite motif protein 63* | *Trim63* | *0.37* | *0.0001* | *1.76* | *0.0252* | *0.80* | *0.2796* |
| *BI284428* | *cullin 1 (predicted)* | *Cul1* | *0.74* | *0.0021* | *1.27* | *0.0379* | *0.94* | *0.5121* |
|  |  |  |  |  |  |  |  |  |
| **GROUP IIB GENES DECREASED IN PBS VS SHAM AND UNCHANGED IN IL6 VS PBS COMPARISON** | | | | | | |  |  |
| NM_013057 | coagulation factor III | F3 | 0.50 | 0.0200 | 1.16 | 0.8863 | 1.31 | 0.3803 |
| AF090695 | CUG triplet repeat, RNA binding protein 2 | Cugbp2 | 0.55 | 0.0043 | 1.19 | 0.6870 | 1.51 | 0.0446 |
| NM_022958 | phosphoinositide-3-kinase, class 3 | Pik3r1 | 0.56 | 0.0366 | 1.42 | 0.4121 | 1.26 | 0.4366 |
| NM_023991 | protein kinase, AMP-activated, alpha 2 catalytic subunit | Prkaa2 | 0.59 | 0.0141 | 1.19 | 0.7028 | 0.92 | 0.7275 |
| AI600029 | activity-dependent neuroprotective protein | Adnp | 0.61 | 0.0139 | 1.30 | 0.3620 | 0.66 | 0.0370 |
| AW521447 | MAD homolog 7 (Drosophila) | Madh7 | 0.63 | 0.0133 | 1.31 | 0.2986 | 0.58 | 0.0060 |
| NM_080895 | Fas apoptotic inhibitory molecule | Faim | 0.65 | 0.0755 | 1.10 | 0.9059 | 0.54 | 0.0158 |
| NM_053687 | schlafen 3 | Slfn3 | 0.70 | 0.0653 | 1.01 | 0.9924 | 20.32 | 0.0000 |
| X02904 | glutathione-S-transferase, pi 1 /// glutathione S-transferase, pi 2 | Gstp1 | 0.71 | 0.0212 | 1.09 | 0.8342 | 0.99 | 0.9335 |
| NM_013135 | RAS p21 protein activator 1 | Rasa1 | 0.72 | 0.0581 | 1.07 | 0.9059 | 0.76 | 0.1217 |
| NM_012555 | v-ets erythroblastosis virus E26 oncogene homolog 1 (avian) | Ets1 | 0.72 | 0.0014 | 0.96 | 0.8880 | 0.43 | 0.0000 |
| BI285959 | Retinoid X receptor alpha | Rxra | 0.72 | 0.0795 | 1.04 | 0.9621 | 0.76 | 0.1314 |
| U30789 | upregulated by 1,25-dihydroxyvitamin D-3 | Txnip | 0.73 | 0.0990 | 1.05 | 0.9557 | 0.90 | 0.6095 |
| AI717081 | Protein phosphatase 2 (formerly 2A), regulatory subunit B (PR 52), alpha isoform | Ppp2r1b | 0.74 | 0.0821 | 1.03 | 0.9689 | 0.92 | 0.6805 |
| BG378230 | mitochondrial ribosomal protein S30 (predicted) | Mrps30 | 0.77 | 0.0043 | 1.20 | 0.1179 | 0.81 | 0.0202 |
| BM385790 | Kruppel-like factor 2 (lung) (predicted) | Klf2 | 0.77 | 0.0691 | 1.36 | 0.1093 | 0.96 | 0.7982 |
| BG673589 | paxillin | Pxn | 0.79 | 0.0642 | 1.20 | 0.3486 | 0.97 | 0.8044 |
| BI285576 | gelsolin | Gsn | 0.79 | 0.0361 | 1.10 | 0.6918 | 0.88 | 0.2655 |
| BI281756 | Parkinson disease (autosomal recessive, early onset) 7 | Park7 | 0.79 | 0.0015 | 1.13 | 0.1619 | 0.94 | 0.3459 |
| BI289543 | zinc finger, MYND domain containing 11 | Zmynd11 | 0.80 | 0.0411 | 1.22 | 0.1973 | 0.84 | 0.1172 |
| AI011448 | Notch gene homolog 2 (Drosophila) | Notch2 | 0.80 | 0.0570 | 1.09 | 0.7551 | 0.59 | 0.0004 |
| BF282337 | integral membrane protein 2B | Itm2b | 0.80 | 0.0529 | 1.05 | 0.9059 | 0.80 | 0.0524 |
| NM_017006 | glucose-6-phosphate dehydrogenase X-linked | G6pdx | 0.81 | 0.0481 | 0.90 | 0.6066 | 0.82 | 0.0772 |
| AI454840 | Phosphodiesterase 1A, calmodulin-dependent | Pde1a | 0.81 | 0.0812 | 1.08 | 0.7978 | 0.96 | 0.7529 |
| AF015718 | interleukin 15 | Il15 | 0.81 | 0.0553 | 1.03 | 0.9434 | 1.41 | 0.0043 |
| NM_012888 | thyroid stimulating hormone receptor | Tshr | 0.83 | 0.0812 | 1.04 | 0.9059 | 0.90 | 0.3332 |
| AF268467 | voltage-dependent anion channel 1 | Vdac1 | 0.84 | 0.0481 | 0.99 | 0.9739 | 0.86 | 0.0937 |
| NM_017165 | glutathione peroxidase 4 | Gpx4 | 0.84 | 0.0402 | 1.15 | 0.2269 | 0.98 | 0.8672 |
| X67108 | brain derived neurotrophic factor | Bdnf | 0.85 | 0.0584 | 1.04 | 0.8880 | 0.93 | 0.4620 |
| AI599419 | Progesterone receptor | Pgr | 0.93 | 0.0642 | 1.02 | 0.8274 | 0.98 | 0.7161 |
| NM_012650 | sex hormone binding globulin | Shbg | 0.94 | 0.0371 | 1.01 | 0.9557 | 0.97 | 0.2344 |
| NM_032074 | insulin receptor substrate 3 | Irs3 | 0.96 | 0.0248 | 1.00 | 0.9783 | 0.91 | 0.0001 |
| NM_012896 | adenosine A3 receptor | Adora3 | 0.96 | 0.0343 | 1.01 | 0.8682 | 0.96 | 0.0249 |
| *NM_080906* | *DNA-damage-inducible transcript 4* | *Ddit4* | *0.39* | *0.0004* | *0.81* | *0.6870* | *0.39* | *0.0007* |
| *AF136231* | *caspase 2* | *Casp2* | *0.44* | *0.0092* | *1.25* | *0.7372* | *0.63* | *0.1279* |
| *BM386306* | *sphingosine kinase 2* | *Sphk2* | *0.62* | *0.0133* | *1.05* | *0.9557* | *0.66* | *0.0348* |
| *AY066016* | *nuclear receptor subfamily 3, group C, member 1* | *Nr3c1* | *0.64* | *0.0385* | *1.19* | *0.7073* | *0.64* | *0.0498* |
| *AA943734* | *mitochondrial protein, 18 kDa* | *MGC94604* | *0.69* | *0.0123* | *1.05* | *0.9103* | *0.54* | *0.0004* |
| *BF283754* | *caspase 7* | *Casp7* | *0.73* | *0.0356* | *0.94* | *0.9059* | *0.73* | *0.0400* |
| *AF051093* | *Bcl-2-related ovarian killer protein* | *Bok* | *0.75* | *0.0129* | *0.99* | *0.9783* | *1.19* | *0.1116* |
| *AA957545* | *T-box 3* | *Tbx3* | *0.77* | *0.0621* | *1.00* | *1.0000* | *0.89* | *0.4593* |
| *BF398331* | *estrogen receptor-binding fragment-associated gene 9* | *Ebag9* | *0.77* | *0.0764* | *1.34* | *0.1343* | *1.41* | *0.0257* |
| *AF262320* | *programmed cell death 8* | *Pdcd8* | *0.79* | *0.0659* | *1.14* | *0.5914* | *0.82* | *0.1292* |
| *NM_019311* | *inositol polyphosphate-5-phosphatase D* | *Inppd5* | *0.80* | *0.0305* | *1.01* | *0.9832* | *1.22* | *0.0524* |
| *NM_021657* | *PH domain and leucine rich repeat protein phosphatase* | *Phlpp* | *0.81* | *0.0503* | *1.01* | *0.9924* | *0.69* | *0.0023* |
| *BI289109* | *max binding protein (predicted)* | *Mnt* | *0.81* | *0.0385* | *1.13* | *0.4866* | *1.99* | *0.0000* |
| *BM391371* | *goliath* | *LOC652955* | *0.81* | *0.0192* | *1.18* | *0.1429* | *0.88* | *0.1251* |
| NM_053401 | nerve growth factor receptor (TNFRSF16) associated protein 1 | Ngfrap1 | 0.82 | 0.0217 | 1.09 | 0.6119 | *0.88* | 0.1562 |
| *NM_022612* | *BCL2-like 11 (apoptosis facilitator)* | *Bcl2l11* | *0.82* | *0.0976* | *1.06* | *0.8880* | *0.82* | *0.1018* |
| BE329013 | SWI/SNF related, matrix associated, actin dependent regulator of chromatin, subfamily a, member 4 | Smarca4 | 0.83 | 0.0342 | 1.17 | 0.1781 | *1.00* | 0.9662 |
| *AF255888* | *SH3-domain kinase binding protein 1* | *Sh3kbp1* | *0.83* | *0.0385* | *1.12* | *0.3780* | *0.80* | *0.0173* |
| *NM_057100* | *growth arrest specific 6* | *Gas6* | *0.86* | *0.0055* | *1.02* | *0.9059* | *0.79* | *0.0002* |
| *BF405951* | *Interleukin-1 receptor-associated kinase 4 (predicted)* | *IL1rk4* | *0.88* | *0.0953* | *1.01* | *0.9832* | *0.85* | *0.0403* |
| *BM390519* | *GULP, engulfment adaptor PTB domain containing 1* | *Gulp1* | *0.89* | *0.0424* | *1.02* | *0.9557* | *0.89* | *0.0488* |
| *AI598730* | *neurotrophin receptor associated death domain* | *Nradd* | *0.92* | *0.0343* | *1.05* | *0.5195* | *0.96* | *0.2926* |
| *AI169601* | *tumor necrosis factor receptor superfamily, member 14 (herpesvirus entry mediator)* | *Tnfrsf14* | *0.94* | *0.0343* | *1.02* | *0.7116* | *0.97* | *0.3440* |
|  |  |  |  |  |  |  |  |  |
| **GROUP III GENES INCREASED IN PBS VS SHAM AND INCREASED IN IL6 VS PBS COMPARISONS** | | | | | | |  |  |
| *NM_012588* | *insulin-like growth factor binding protein 3* | *Igfbp3* | *1.88* | *0.0317* | *2.2114613* | *0.0311* | *1.39* | *0.2698* |

^A^Genes listed in regular type are anti-apoptotic, while genes listed in italics are pro-apoptotic.

^B^FDR: False discovery rate.
